# High-Resolution Mapping of a Genetic Locus Regulating Preferential Carbohydrate Intake, Total Kilocalories, and Food Volume on Mouse Chromosome 17

**DOI:** 10.1371/journal.pone.0110424

**Published:** 2014-10-20

**Authors:** Rodrigo Gularte-Mérida, Lisa M. DiCarlo, Ginger Robertson, Jacob Simon, William D. Johnson, Claudia Kappen, Juan F. Medrano, Brenda K. Richards

**Affiliations:** 1 Department of Animal Science, University of California Davis, Davis, California, United States of America; 2 Genetics of Eating Behavior Laboratory, Pennington Biomedical Research Center, Louisiana State University System, Baton Rouge, Louisiana, United States of America; 3 Department of Developmental Biology, Pennington Biomedical Research Center, Louisiana State University System, Baton Rouge, Louisiana, United States of America; 4 Biostatistics Department, Pennington Biomedical Research Center, Louisiana State University System, Baton Rouge, Louisiana, United States of America; Nanjing Forestry University, China

## Abstract

The specific genes regulating the quantitative variation in macronutrient preference and food intake are virtually unknown. We fine mapped a previously identified mouse chromosome 17 region harboring quantitative trait loci (QTL) with large effects on preferential macronutrient intake-carbohydrate (*Mnic1*), total kilcalories (*Kcal2*), and total food volume (*Tfv1*) using interval-specific strains. These loci were isolated in the [C57BL/6J.CAST/EiJ-17.1*-(D17Mit19*-*D17Mit50)*; B6.CAST-17.1] strain, possessing a ∼40.1 Mb region of CAST DNA on the B6 genome. In a macronutrient selection paradigm, the B6.CAST-17.1 subcongenic mice eat 30% more calories from the carbohydrate-rich diet, ∼10% more total calories, and ∼9% more total food volume per body weight. In the current study, a cross between carbohydrate-preferring B6.CAST-17.1 and fat-preferring, inbred B6 mice was used to generate a subcongenic-derived F_2_ mapping population; genotypes were determined using a high-density, custom SNP panel. Genetic linkage analysis substantially reduced the 95% confidence interval for *Mnic1* (encompassing *Kcal2* and *Tfv1*) from 40.1 to 29.5 Mb and more precisely established its boundaries. Notably, no genetic linkage for self-selected fat intake was detected, underscoring the carbohydrate-specific effect of this locus. A second key finding was the separation of two energy balance QTLs: *Mnic1/Kcal2/Tfv1* for food intake and a newly discovered locus regulating short term body weight gain. The *Mnic1/Kcal2/Tfv1* QTL was further de-limited to 19.0 Mb, based on the absence of nutrient intake phenotypes in subcongenic HQ17IIa mice. Analyses of available sequence data and gene ontologies, along with comprehensive expression profiling in the hypothalamus of non-recombinant, *cast/cast* and *b6/b6* F_2_ controls, focused our attention on candidates within the QTL interval. *Zfp811*, *Zfp870*, and *Btnl6* showed differential expression and also contain stop codons, but have no known biology related to food intake regulation. The genes *Decr2*, *Ppard* and *Agapt1* are more appealing candidates because of their involvement in lipid metabolism and down-regulation in carbohydrate-preferring animals.

## Introduction

Genes influencing the quantitative variation in caloric and macronutrient-specific intakes, which can predispose individuals to obesity and obesity-related diseases, are virtually unknown [1], [2]. Whole-genome linkage analysis is the most commonly used approach for discovering the genetic contribution to eating behavior [3]. In humans, genome-wide linkage analyses have identified several regions of significant linkage for macronutrient intake [4]–[7], e.g., for energy and macronutrient intakes on human chromosome 3q27.3 [6] which corresponds to the region between 22,776,516 and 22,776,729 bp on mouse chromosome (MMU) 16 (GRCh37/hg19 assembly, UCSC). To our knowledge, none of the quantitative trait loci (QTL) found in humans correspond to those identified in mice, with the exception of a locus for total energy on human chromosome 20q13.13 [7] that overlaps *Kcal3*, a body weight-dependent QTL for kilocalorie intake on MMU2 [8]. Mice are excellent models for genetic mapping because interval-specific strains can be bred to isolate and test loci associated with specific traits. Another key advantage of using laboratory animals for studies of eating behavior instead of humans [9] is the ability to provide a controlled environment, including manipulation of the diet and accurate measurement of food consumption.

Despite its importance in energy balance and thus the obesity equation, very few studies have identified QTL for energy intake in mammals. In mice, Allan *et al.* [10] found eight loci influencing energy consumption on five chromosomes by recording chow intake at weekly intervals. Mathes *et al.* [11] detected a QTL on MMU12 for chow intake during exercise that was determined by the C57BL/6J-derived allele. We and others have shown that mice, similar to people, exhibit a wide range in the amounts of carbohydrate and fat eaten when selecting among food choices [12]–[19], providing evidence for naturally occurring variation. We previously investigated the genetic basis for total energy and macronutrient-specific intakes in F_2_ progeny derived from an intercross of carbohydrate-preferring CAST/EiJ (CAST) and fat-preferring C57BL/6J (B6) mouse inbred strains [14]. We identified six significant and two suggestive QTL for self-selected macronutrient intake (fat-rich *vs.* carbohydrate-rich diets), as well as three significant loci for total kilocalorie intake [8], providing strong evidence for multiple genetic determinants of food intake. In particular, highly significant QTL for macronutrient intake-carbohydrate (*Mnic1)* and total kilocalorie intake (*Kcal2*) were uncovered on proximal MMU17. *Mnic1* was consistent with the parental strain phenotype, i.e., CAST alleles at *Mnic1* increased the preferential intake of carbohydrate over fat, independent of body weight.

The *Mnic1* and *Kcal2* QTLs were successfully captured in both congenic and subcongenic strains, eventually narrowing the interval to a 40.1 Mb region [20]–[22]. The subcongenic strain B6.CAST-*D17Mit19-D17Mit50* (referred to here as B6.CAST-17.1) possesses CAST genetic material between 4.8 and 45.4 Mb of MMU17 on an otherwise B6 genome. Congenic strains, by design, eliminate genetic variation outside the locus of interest, and thus can establish whether the locus of interest acts independently to exert its phenotypic effects. In a diet choice paradigm, the B6.CAST-17.1 subcongenic mice ate 30% more carbohydrate-rich diet and 10% more total calories per gram body weight, yet consumed similar amounts of the fat-rich diet when compared to B6 littermates [20], demonstrating that the MMU17 subcongenic interval retained and confirmed the effects of *Mnic1* and *Kcal2*. In accordance with the higher volume/weight and lower calorie density of carbohydrate relative to fat, the B6.CAST-17.1 strain also consumed a larger food volume, consistent with a QTL for total food volume (*Tfv1*) that we identified in the same genomic region [23].

To shorten the responsible interval and thereby reduce the number of candidate genes, we have now performed single nucleotide polymorphism- (SNP-) based fine mapping of the *Mnic1/Kcal2/Tfv1* QTL using an interval-specific congenic F_2_ mapping population. To further de-limit the causative interval, we also evaluated the macronutrient diet selection and kcal/g intake phenotypes of an existing subcongenic strain (C57BL/6J*^hg/hg^*.CAST-(*rs49640908*–*rs47909630*). In addition to the fine mapping strategies, we analyzed bioinformatics data, including available sequence, and generated gene expression data from a trait-relevant tissue–hypothalamus–to highlight potential candidate genes. Genes uncovered using both approaches have been proposed as likely causative genes underlying a QTL [24].

## Materials and Methods

### Mouse lines and breeding strategy

**Figure 1:**
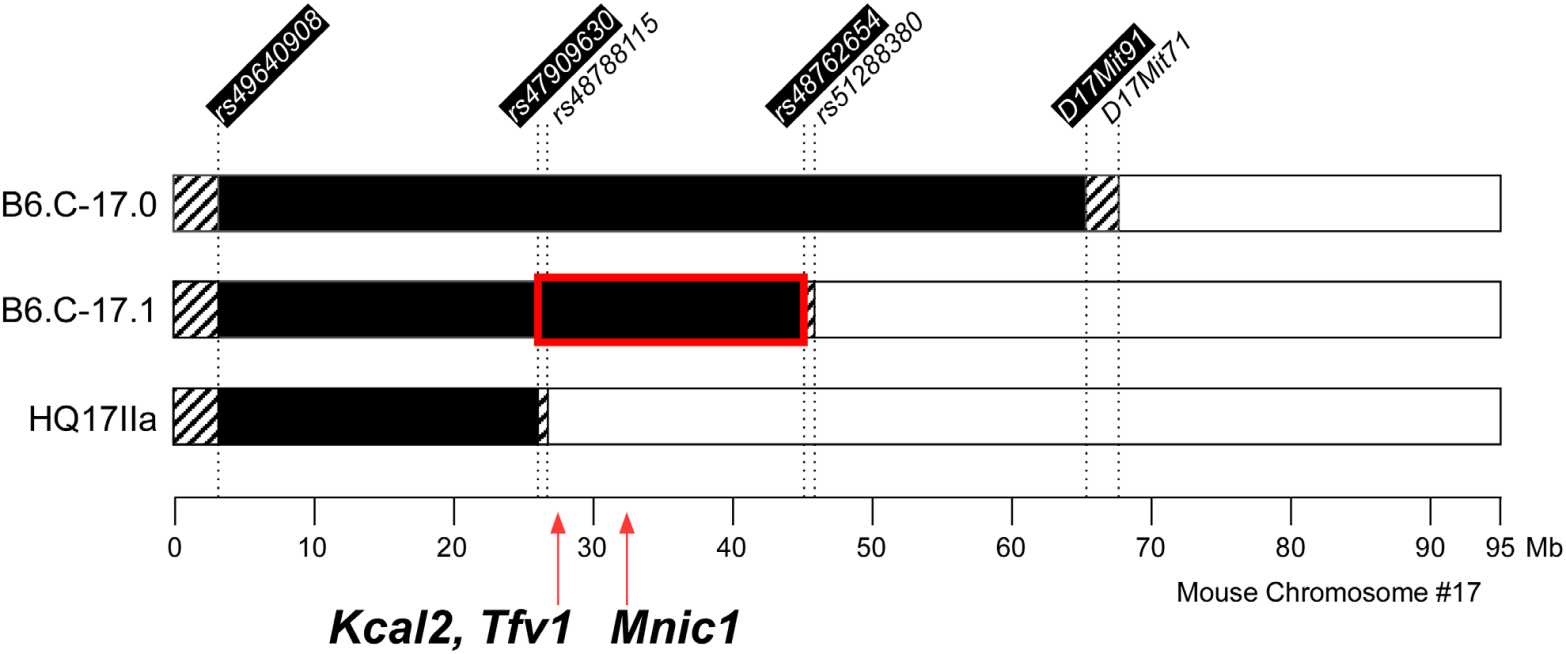
The critical *Mnic1/Kcal1*/*Tfv1* QTL region on mouse chromosome 17 was reduced to 19.0 Mb. Legend: Congenic and subcongenic strains with CAST/EiJ alleles introgressed on the wild type C57BL/6J (B6) or mutant C57BL/6J-*^hg/hg^* genome are illustrated. Solid bars indicate CAST donor regions, open bars indicate B6 genotype, and hatched bars designate intervals of undetermined genotype, as defined by SNP or Mit markers (top). The fine-mapped interval encompassing carbohydrate-specific macronutrient intake (*Mnic1*; peak at 32.49 Mb), total kilocalories (*Kcal1*; peak at 27.19 Mb) and total food volume (*Tfv1*; peak at 27.10) is specified by the bar outlined in red.

The development of the B6.CAST-17.0 speed congenic and B6.CAST-17.1 subcongenic strains has been described previously [20]–[22]. The MMU17 subcongenic interval was previously defined as a 40.6 Mb CAST minimal donor region extending from *D17Mit19* to *D17Mit50* [20]. In the current study, this region was more precisely defined as a 42.5 Mb interval bounded by SNP markers *rs49640908* (proximal) and *rs48762654* (distal) (Figure 1). For mapping purposes, a congenic-by-recipient F_2_ population was generated by mating a single B6.CAST-17.1 congenic male to multiple C57BL/6J (stock #000664) females purchased from The Jackson Laboratory (Bar Harbor, ME). The resulting F_1_ mice were intercrossed and in this manner, a large cohort of B6.CAST-17.1 congenic F_2_ was generated. To further map the causative interval on proximal chromosome 17, we also phenotyped subcongenic (n = 14) and wildtype (n = 14) mice from the C57BL/6J*^hg/hg^*.CAST(*rs49640908–rs47909630*) line (referred to as HQ17IIa). This HQ17IIa subcongenic mouse strain, developed in the laboratory of Dr. Juan F. Medrano at the University of California, Davis, is homozygous for CAST/EiJ (CAST) alleles from 3.19 to 26.08 Mb on MMU 17 on a *high growth* background (Figure 1). *High growth* mice (C57BL/6J*^hg/hg^*) show an increased body weight without obesity due to the high growth (*hg*) deletion which eliminates expression of the *Socs2* (suppressor of cytokine signaling 2), a negative regulator of growth hormone signaling [25].

All animals were bred and reared in polycarbonate cages on sterilized corncob bedding, and maintained under controlled conditions of temperature (21±2°C), humidity (30–70%), and lighting (14 h light:10 h dark). Mice were fed standard chow diet (LabDiet 5001; 28% protein, 12% fat, and 60% carbohydrate kcal/g) and tap water ad libitum. Pups were weaned at 24–26 days of age and housed with siblings in same-sex groups of 1–4. Only progeny from litters containing 4–10 pups were used for breeding and experiments, and the average litter size was 7.48 pups. All animal husbandry protocols were in accordance with the guidelines of the American Association for Accreditation of Laboratory Animal Care (AAALAC). All experimental protocols conducted in this study were approved by the Pennington Biomedical Research Center Institutional Animal Care and Use Committee.

### Experimental design

The general experimental approach used to fine map the MMU 17 QTL involved phenotyping and genotyping male F_2_ mice possessing at least one recombination within the subcongenic region (n = 168), as well as their non-recombinant F_2_ littermates, i.e., *cast/cast* (n = 44), *b6/b6* (n = 47), and *cast/b6* (n = 60), for a total of 319 congenic F_2_. These numbers were based on power calculations which determined that ∼170 animals with at least one recombination within the subcongenic region were needed to detect a significant difference in carbohydrate/protein (C/P; see below) kcal at 80% power. A variety of body weight, body composition, food intake, and organ weight phenotypes were assessed in five experimental cohorts of randomly selected, age contemporary male F_2_ mice. Standardized phenotyping methods have been previously described in detail [8]. Briefly, 10–16 week old F_2_ mice were first weighed and then housed individually in a hanging, stainless steel mouse cage with wire mesh floor, containing a polyvinylchloride tube for nesting. To minimize heat loss via convection in these cages, animals were maintained at an ambient temperature of 26–27°C. Before being presented with a choice between macronutrient diets, animals were allowed a minimum of 7 d to adapt to the special housing conditions, as evidenced by a return to baseline body weight. When not tested in the macronutrient diet selection protocol, mice were housed in polycarbonate cages and fed standard chow *ad libitum* (LabDiet No. 5001, Richmond, IN).

### Measurement of phenotypes

Body weights and body composition were measured twice: immediately before and after the 10 d diet selection study. Nuclear magnetic resonance (NMR) with a Bruker Mice Minispec MQ10 series NMR analyzer (Bruker Optics, Inc., Billerica, MA) was used to provide estimates of body fat, lean body tissue, and fluid in awake animals. This instrument was calibrated daily according to the manufacturer’s instructions. Intakes of fat/protein, carbohydrate/protein, and total calories were determined in singly-housed, adult male mice, using a macronutrient diet protocol. Specifically, for 10 days mice were provided a choice between fat/protein (F/P) and carbohydrate/protein (C/P) diet mixtures, equivalent for protein (22% of energy) and with the balance of calories contributed by either fat or carbohydrate (78%). Both diets contained casein (protein), minerals and vitamins. The C/P diet was composed of corn starch and powdered sucrose, whereas the F/P diet contained vegetable shortening from one of two sources, according to availability. The experimental diet composition and complete details of the phenotyping procedures have been described previously [8]. Briefly, each diet was presented in a custom two oz. glass jar with stainless steel lid (Unifab, Kalamazoo, MI); food intake and all spillage were measured daily to the nearest 0.1 g. Following the 10 d diet self-selection protocol, animals were returned to a standard chow diet for at least two weeks. Two days before euthanasia, animals were again provided access to the macronutrient selection diets. The mice were fasted for 4 h prior to euthanasia, which consisted of an overdose of isoflurane gas inhalation followed by decapitation. Bilateral epididymal (EPI) fat pads and whole liver were dissected and wet weights were determined. The hypothalamus was carefully lifted from the ventral surface of the brain based on visual localization, e.g., relative to the optic chiasm and pituitary. Spleens were collected for DNA isolation.

### Genotyping and SNP selection

Genomic DNA was isolated from spleens using phenol/chloroform extraction and suspended in TE buffer (10 mM Tris, pH 7.5, 1 mM EDTA) at a final concentration of 50 ng/µl, as determined by Quant-iT PicoGreen (Invitrogen). To perform QTL analysis, a custom SNP panel was designed based on the Perlegen SNP dataset available at the Mouse Phenome database (http://phenome.jax.org/). We identified a total of 384 polymorphic markers between CAST/EiJ and C57BL/6J, located from 3.18 to 47.1 Mb on MMU 17. SNP were selected at uniform intervals, giving preference to those located within coding regions and previous QTL locations (Table S1), resulting in an average spacing of 0.114±0.066 Mb across the interval. This SNP panel containing 301 markers (Table S1) was used to genotype 319 mice from the B6.CAST-17.1 F_2_ intercross, and additional controls, using the Illumina GoldenGate assay and VeraCode technology on the BeadXpress platform. Genotype calls were carried out using Illumina Genome Studio software. The genotype call rate averaged 98.7% for the DNA samples and 47 control samples showed complete genotype concordance.

### Fine-mapping of QTL for body composition, macronutrient diet selection and total food intake (kcal, g)

Measurements of daily consumption of fat/protein (F/P) kcal, carbohydrate/protein (C/P) kcal, total kcal and total gram intake were expressed as 10 d sums as previously described [8]. Gain in body weight and body composition components (lean mass, fat mass, fluid mass) was defined as the difference between the ending (e.g., BW2) and starting values (e.g., BW1) over the 10 d of macronutrient diet selection. Gain was also determined for the difference between end-of-study body weight (BW3) and earlier weights (BW1, BW2). Phenotypic differences among F_2_ non-recombinant controls, possessing *cast/cast*, *b6/cast*, and *b6/b6* genotypes across the subcongenic segment, were estimated using a mixed effects, general linear statistical model (*Proc MIXED, SAS v9.3*) to perform a repeated measures analysis of variance (covariance) while accounting for the effects of fat source, initial age, baseline body weight and baseline lean mass. These covariates were considered to be the most biologically and experimentally relevant, e.g., lean body mass is metabolically active and contributes significantly to energy expenditure [26]. Significance of specific discrepancies among genotypes was tested using orthogonal contrasts on the three genotypes (*b6/b6, b6/cast, cast/cast*). Relevant outcomes were summarized in either tables or graphs as covariate-adjusted (least squares) mean ± standard error of mean (SEM). Pearson correlation coefficients among dependent variables were determined and are reported for four categories of phenotypes, including food intake, body composition, body weight, and tissue end weights.

### Statistical genetic analyses

Linkage analysis was carried out using both interval mapping and single marker analysis. Interval mapping was performed using the scanone function with the Halley-Knott regression method and imputation of pseudomarkers at 0.01 Mb from the R/qtl package of R: Language and Environment [27], [28]. Fat source, initial age (age1), baseline body weight (BW 1), and initial lean body mass (NMR Lean 1) were included as additive covariates as described above. To define the genome-wide LOD score threshold required as significant or suggestive for each specific trait, genome-wide significance levels were estimated by using 1,000 permutations with the n.perm argument in scanone [29]. Ninety-five percent confidence intervals (CI) were determined by the bayesint function in R/qtl. To estimate the QTL effect, genotypes were coded as 1, 2, and 3 for b6/b6, b6/cast and cast/cast, respectively. The genotype coefficient was considered as the estimate of the additive effect of the cast allele. Single marker analysis was carried out at each individual SNP using a general linear model (glm function) that included fat source, initial age, baseline body weight and initial lean body mass as covariates. These results were similar to those from interval mapping, and therefore are not presented.

The results of single QTL analysis suggested the presence of multiple QTL segregating in the congenic strain, thus we tested for the presence of multiple QTL using the *stepwiseqtl* function in R/qtl [30]. To increase the power to detect multiple QTL a combined cross analysis was performed by combining the genotype and phenotype results from two separate mapping populations [31]: C57BL/6J×B6.CAST-17.1 subcongenic (n = 319 F_2_) in the current study, and our previous C57BL/6J×CAST/EiJ strain intercross (n = 502 F_2_) [8]. The results of this combined cross analysis provided insufficient evidence to support the hypothesis of multiple QTL in the region.

### Experimental design for gene expression analyses

Global gene expression profiles in the hypothalamus were compared between non-recombinant subcongenic-derived F_2_ mice, possessing a genotype of *cast/cast* (n = 12) or *b6/b6* (n = 12) across the Chr 17 subcongenic segment and *b6/b6* across the rest of the genome. RNA was isolated from individual mice of each genotype that were selected for study. Specifically, twelve mice of each genotype that displayed the most divergent phenotypic values for self-selected carbohydrate and total kcal intake were chosen for gene expression analysis, i.e., *cast/cast* mice with the highest and *b6/b6* mice with the lowest kcal intake from carbohydrate, or the 25% tails of the distribution. Tissues were harvested ∼48 h after re-initiation of the carbohydrate/protein *vs*. fat/protein diets, following an extended wash-out period on chow diet after completion of the 10 d macronutrient selection test (see Methods above). The hypothalamus was selected for study based on its key role in the behavioral and metabolic control of food intake [32].

### Gene expression profiling

Total RNA from the hypothalamus was extracted and purified using an AllPrep RNA/Protein kit (Qiagen, Valencia, CA) and re-suspended in Nanopure water. RNA quality was assessed by electrophoresis using an Agilent 2100 Bioanalyzer (Total RNA Nano; Agilent) and all samples showed an RIN number (RNA integrity number) of ≥7.0. Gene expression profiling was performed by serial analysis of gene expression followed by high-throughput sequencing (SAGE-Seq) using the 5500XL SOLiD system (Life Technologies). Briefly, SAGE libraries containing 27-bp, 3′ tags for all transcripts within a sample were constructed using a SOLiD SAGE kit (Life Technologies), at the PBRC Genomics Core Facility. Each library was then labeled with a unique barcode sequence and high-throughput sequencing was performed using SOLiD technology (Life Technologies). Sequence mapping was performed using a modified version of the SOLiD SAGE Analysis Software v1.10 (Life Technologies) and defined analysis parameters. Sequenced reads were aligned using the mouse transcriptome RefSeq mRNA database and genome build GRCm38/mm10. Tag hits, i.e., successfully aligned reads, were normalized according to DESeq by estimating the size factor for each sample and dividing the sample counts by the corresponding size factor. Differential expression analysis was performed using the DESeq R/Bioconductor package [33]. Genes with significantly altered expression in the *cast/cast* congenic F_2_ samples compared to that of *b6/b6* congenic F_2_ were stringently defined by a fold change of ≥1.5 and a *P* value of ≤0.01. Two-way hierarchical clustering, by means of Ward’s minimum variance criterion method, was applied to normalized and standardized expression data using the tools in JMP Genomics, Version 10 (SAS Institute Inc., Cary, NC). The SAGE-Seq analyses reported here have been deposited to GEO (http://ncbi.nlm.nih.gov/geo/; accession number GSE60756).

### Gene annotation

Biological process annotation was performed using the WEB-based Gene SeT AnaLysis Toolkit (WebGestalt; http://bioinfo.vanderbilt.edu/webgestalt/) which draws on a number of centrally and publicly curated databases including Gene Ontology (GO) and KEGG (Kyoto Encyclopedia of Genes and Genomes) [34]. The results of this analysis are illustrated in Figure S3. For the enrichment calculations in WebGestalt (Figure S4), we executed a Gene Ontology analysis against a background of all mouse RefSeq genes, using the default settings for classification stringency. The significance of gene enrichment was based on *P*-values generated by the hypergeometric test, adjusted for multiple testing. Gene annotation categories were considered to be significantly overrepresented if the associated *P* value was less than 0.05.

## Results

### Phenotypes in non-recombinant subcongenic-derived-F_2_ mice

Macronutrient and total calorie intakes, body weight, and body composition were measured in non-recombinant subcongenic-derived F_2_ mice (see Table 1). The mean kcal intake selected from carbohydrate/protein (C/P) diet was ∼25% higher in *cast/cast* F_2_ compared to *b6/b6* F_2_ mice, thus confirming and extending previous observations [20], [21]. Based on least squares means, over-dominance, i.e., the mean of the *b6/cast* hybrid population as being less or more than the average value of the two parental strains [35], was observed for eight phenotypes (Table 1). Both the *cast/cast* F_2_ and *b6/cast* F_2_ mice consumed more total kcal from carbohydrate and less from fat compared to *b6/b6* F_2_ littermates (*P*<0.005), indicating a dominant effect of the *cast* allele. The cumulative intake of total kilocalories for 10 d was also higher in non-recombinant *cast/cast* compared to *b6/b6* (*P*<0.005) F_2_ mice. A dominant effect of the *cast* allele on cumulative total food volume (*P*<0.05) was observed, which may reflect higher consumption of the less-energy-dense carbohydrate/protein diet (3.61 kcal/g), than the fat/protein diet (5.96 kcal/g), as indicated by the strong positive correlation between C/P kcal and food volume (*r* = 0.87, *P*<0.0001; Table S4). In non-recombinant F_2_ mice, short-term weight gain was positively associated with total calorie intake (*r* = 0.51, *P*<0.0001) and, to some extent, fat calories (*r* = 0.22, *P*<0.01), but not carbohydrate (*r* = 0.12, *P* = NS) (Table S5).

**Table 1:** Phenotypic data for food intake, body weight, body composition and organ weights in non-recombinant B6.CAST-17.1-derived F_2_ mice.

	B/B (n = 47)	B/C (n = 60)	C/C (n = 44)
10d sum C/P kcal^a^^,^ b	52.9±2.6^§^	67.6±2.5**	70.5±2.9
10d sum F/P kcal^a^^,^ b	55.9±2.5*	47.6±2.3*	43.6±2.7
10d sum Total kcal^a^	108.8±1.5**	115.2±1.4	114.1±1.7
10d sum Total g^a^^,^ b	24.0±0.5§	26.7±0.4*	26.9±0.5
Baseline BW 1 (g)	23.7±0.1	23.8±0.1	23.9±0.1
BW 2 after 10 d diet (g)	25.0±0.1	25.2±0.1	25.4±0.2
BW3 - dissection (g)^a^^,^ b	25.7±0.1**	26.2±0.1*	26.3±0.1
BW gain (2 minus 1)	1.2±0.1	1.4±0.1	1.6±0.2
NMR fat 1	1.85±.05	1.81±0.05	1.97±0.06
NMR fat 2	2.58±0.12	2.70±0.11	2.88±0.13
NMR fat gain	0.73±0.10	0.89±0.10	0.92±0.11
NMR lean 1^b^	18.30±0.06	18.19±0.06*	18.02±0.07
NMR lean 2^b^	18.56±0.10	18.58±0.09*	18.86±0.11
NMR lean gain^b^	0.25±0.11	0.39±0.11**	0.84±0.12
NMR fluid 1	0.68±0.02	0.66±0.02	0.66±0.02
NMR fluid 2^a^	0.76±0.02**	0.66±0.02	0.69±0.02
NMR fluid gain^a^	0.08±0.02*	0.00±0.02	0.03±0.03
EPI fat pad weight (g)	0.350±0.013	0.380±0.012	0.375±0.014
Liver weight (g)	1.405±0.027	1.467±0.025	1.462±0.029

Values indicate least squares means ± SE.

a Difference between B/B and C/C; probability value indexed in B/B column.

b Difference between the B/C population and the average of its two parental strains combined, adjusted for fat source, initial age, baseline body weight, and baseline lean mass; probability value indexed in B/C column.

*P<0.05; **P<0.005; ^§^ *P*<0.0001. Carbohydrate/protein (C/P) and fat/protein (F/P) diets were presented simultaneously for 10 days. BW, body weight; NMR, nuclear magnetic resonance; EPI, epididymal.

**Figure 2:**
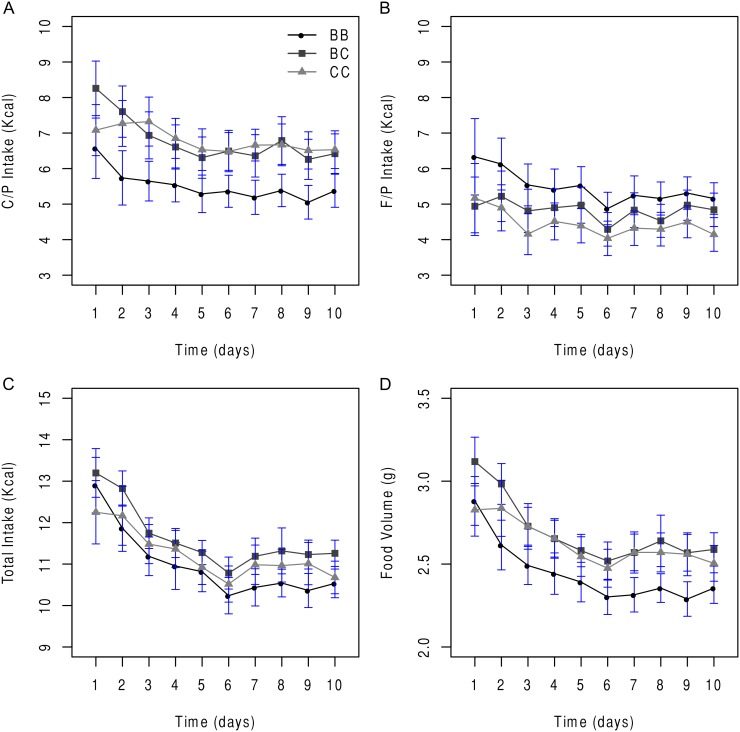
Non-recombinant B6.CAST-17.1 F_2_ mice exhibit increased intake of carbohydrate kcal, total kcal and total food volume. Legend: Daily consumption of (A) carbohydrate/protein kcal (C/P) versus (B) fat/protein kcal (F/P), total kcal (C) and total food volume (D) in non-recombinant B6.CAST-17.1 F_2_ mice. Values are mean ± SE. Relative to *b6/b6* F_2_, both *cast/cast* (*P*<0.005, Tukey-Kramer) and *b6/cast* (*P*<0.001) F_2_ mice consumed more C/P kcal (A), with the exception of *day 1* when *b6/cast*>*b6/b6* F_2_ mice = *cast/cast* F_2_ [genotype by day: *F* (18, 1187) = 2.16, *P*<0.003]. By contrast, the *b6/b6* F_2_ ate more F/P kcal when compared to *cast/cast* F_2_ (*P*<0.008) but not *b6/cast* F_2_ (*P* = 0.10) (B), during the 10 d study. With respect to total kcal, all genotypes displayed a pronounced hyperphagic response to the diets at the beginning of the study, which subsided gradually over time [day: *F* (9, 938) = 21.10, *P*<0.0001], independent of genotype [genotype×day: *F* (18, 938) = 0.94, *P* = 0.53]. Relative to *b6/b6* F_2_, both *cast/cast* (*P*<0.005) and *b6/cast* (*P*<0.0001) F_2_ mice consumed more total g over 10 d.

The 10 d time-course of genotype effects on C/P versus F/P intake in non-recombinant subcongenic F_2_ animals is illustrated in Figure 2. Relative to the *b6/b6* genotype, both *cast/cast* (*P*<0.005, Tukey-Kramer) and *b6/cast* (*P*<0.001) F_2_ mice consumed more C/P kcal (Figure 2A), with the exception of *day 1* when *b6/cast*>*b6/b6* F_2_ mice = *cast/cast* F_2_ [genotype by day: *F* (18, 1187) = 2.16, *P*<0.003]. By contrast, the *b6/b6* F_2_ ate more F/P kcal over 10 d, when compared to *cast/cast* F_2_ (*P*<0.008) but not *b6/cast* F_2_ (*P* = 0.10) (Figure 2B). Based on both total calorie and g consumption, the animals displayed a pronounced hyperphagic response to the novel diets at the beginning of the study, which gradually subsided over time [e.g., total kcal, day: *F* (9, 938) = 21.10, *P*<0.0001], independent of genotype [genotype×day: *F* (18, 938) = 0.94, *P* = 0.53]. Relative to the *b6/b6* genotype, non-recombinant F_2_ with the *cast/cast* (*P*<0.005) and *b6/cast* (*P*<0.0001) genotype consumed more total g over 10 d.

Both body weight and body fat content of the non-recombinant, littermate F_2_ control mice were similar among genotypes at baseline (Table 1). After 10 d of macronutrient diet selection, all three genotypes gained similar amounts of body weight (∼5–6%) and body fat (28–32%). By the end of the entire study period (BW3), both the *cast/cast* F_2_ and *b6/cast* F_2_ mice had gained a small (∼9%) but significantly greater amount of weight compared to *b6/b6* F_2_ littermates (*P*<0.005; Table 1). There were no genotype effects on epididymal fat pad or liver weights.

### High resolution mapping of *Mnic1*, *Kcal2* and *Tfv1* QTL

**Figure 3:**
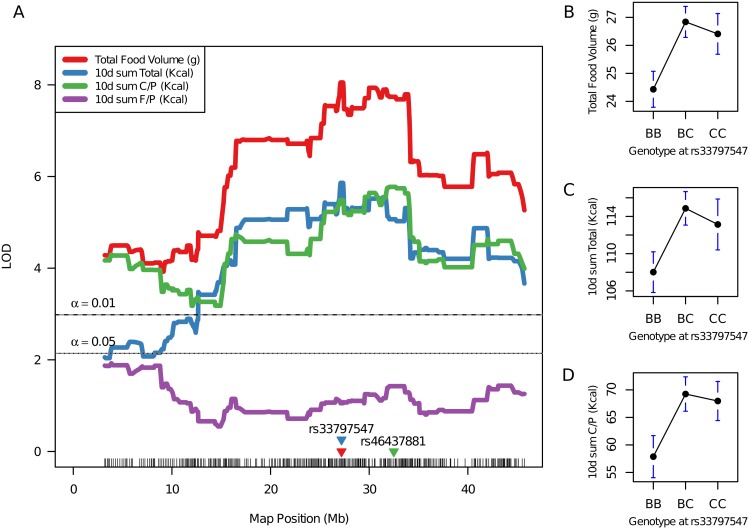
Preferential carbohydrate and total food intake is controlled by *Mnic1* and *Kcal/Tfv1* loci, respectively. Legend: Interval plot of proximal Chr 17 illustrates fine-mapped QTL for carbohydrate intake (*Mnic1*), total calorie intake (*Kcal2*) and total food volume consumed (*Tfv1*) in B6.CAST-17.1 subcongenic F_2_ mice (left) and uncorrected phenotypic means for the peak marker of each QTL (right). Significance threshold values established for α = 0.01 and α = 0.05 were LOD scores of 2.98 and 2.14, respectively, as determined by 1,000 permutation tests. On the interval map, locations of markers nearest the peak LOD scores are shown on the x-axis, e.g., *rs33797547*. On the right, genotype effect plots at marker *rs33797547* show the CAST (CC) allele is associated with a higher intake of g, total kcal, and carbohydrate/protein kcal compared to the B6 (BB) allele, and the F1 (BC) responses for all three phenotypes resembled those of the CC, indicating dominance of the CAST allele. Values are mean ± SEM. See Table 2 for a list of all QTLs.

A subcongenic-derived intercross was constructed to reduce the QTL influencing preferential carbohydrate intake, total calories and food volume to a smaller region containing fewer genes. Compared with a whole-genome scan, this approach to linkage analysis provides a substantial increase in statistical power to detect and resolve QTL due to the elimination of background genetic noise. As a result, we narrowed and more precisely localized the QTL for macronutrient intake-carbohydrate (*Mnic1*) (*rs46437881*, 32.49 Mb; LOD = 5.77) (Table 2). In addition, the peak position of co-localized QTL for total kilocalorie intake (*Kcal2*) and total food volume (*Tfv1*) was localized to 27.19 Mb (*rs33797547*), with highly significant LOD scores of 5.86 and 8.05, respectively (Table 2; Figure 3). No significant linkage for F/P kcal intake was detected (LOD = 1.92) in this subcongenic interval. These results clearly replicated, yet with far greater mapping resolution, the Chr 17 QTL identified in the original F_2_ intercross [8]. Specifically, the peak locus for *Kcal2* and *Tfv1* (*rs33797547*), was fine mapped to ∼27.1 Mb (*rs33797547*). Positioned ∼5.30 Mb distal to this locus, is the peak for *Mnic1* (*rs46437881*), suggesting that a minimum of two loci are responsible, for both the amount of food consumed in a diet choice paradigm and the macronutrient composition of that food. Sub-peaks within the CI suggest the possibility of more than one genetic element that is contributing to the major phenotypic effects of these QTL [36]. An examination of The Jackson Laboratory QTL database (www.informatics.jax.org) revealed a total of 37 documented mouse QTL within the 15.55–45.12 Mb CI for *Mnic1,* as determined by linkage analysis. Only two of these QTL are obesity-related, i.e., *Obrq13* [37] and *Wta4* [38], and none pertain to macronutrient preference, energy intake, or food consumption, with the exception of *Mnic1*, *Kcal2*, and *Tfv1* [8], [23].

**Table 2:** QTLs for nutrient intake, body weight, and body composition in B6.CAST-17.1-derived F_2_ mice.

*QTL name*	*Phenotype*	*Peak (Mb) (Marker)*	*LOD*	*95% CI (Mb) (Markers)*	*Effect of CAST allele*
*Tfv1*	10d sum Totalfood volume (g)	27.19 (*rs33797547*)	8.05	16.41–34.18 (*rs33750505–rs46967024*)	+1.30±0.25***
*Kcal2*	10d sum Total(Kcal)	27.19 (*rs33797547*)	5.86	16.41–42.12 (*rs33750505–rs33341718*)	+3.61±0.81***
*Mnic1*	10d sum C/P (Kcal)	32.49 (*rs46437881*)	5.77	15.55–45.12 (*rs48970296–rs46074101*)	+6.87±1.40***
----	NMR fat gain (g)	21.74 (*rs48587745*)	3.46	10.49–41.83 (*rs33750661–rs47337390*)	+0.17±0.05**
----	BW 2 (g)	11.06 (*rs33738432*)	3.45	3.19–31.84 (*rs49640908–rs45936635*)	+0.31±0.08***
----	NMR 2 fat (g)	16.53 (*rs33747676*)	3.18	3.19–37.64 (*rs49640908–rs33721754*)	+0.21±0.06***
----	EPI Weight (g)	21.31 (*rs51776929*)	3.08	5.44–38.20 (*rs48030175–rs33809121*)	+0.022±0.006***
----	NMR 2 lean (g)	31.44 (*rs51849267*)	2.48	9.14–45.50 (*rs46011679–rs49209168*)	+0.19±0.05**
----	BW 3 (g)	45.12 (*rs46074101*)	2.07	6.73–45.73 (*rs33695293–rs48762654*)	+0.27±0.08***
----	10d sum F/P(Kcal)	3.86 (*rs33651689*)	1.92	3.19–45.73 (*rs49640908–rs48762654*)	−2.44±1.26^§^
----	NMR 2 free fluid(g)	5.89 (*rs47725817*)	1.60	3.19–45.73 (*rs49640908–rs48762654*)	−0.01±0.01
----	Liver Weight (g)	8.17 (*rs48213049*)	1.23	3.19–45.73 (*rs49640908–rs48762654*)	−0.02±0.01
----	NMR 1 fat (g)	33.79 (*rs33219958*)	1.14	3.19–45.73 (*rs49640908–rs48762654*)	0.02±0.03
----	NMR 1 free fluid(g)	45.73 (*rs48762654*)	0.97	11.53–45.73 (*rs33653408–rs48762654*)	−0.01±0.01

Genome coordinates are based on the Genome Reference Consortium Mouse Build 38 (GRCm38). Mb = megabase. LOD = logarithm (base 10) of odds. Significant linkage (*P*<0.01 and *P*<0.05) was defined as LOD>2.98 and LOD>2.14 respectively, based on 1,000 permutations. “----”, un-named. CI = confidence interval. C/P = carbohydrate/protein; BW = body weight. NMR = nuclear magnetic resonance. F/P = fat/protein. EPI = epididymal fat. ***P≤0.0001; **P≤0.001; *P≤0.01; ^§^ *P*≤0.05.

**Figure 4:**
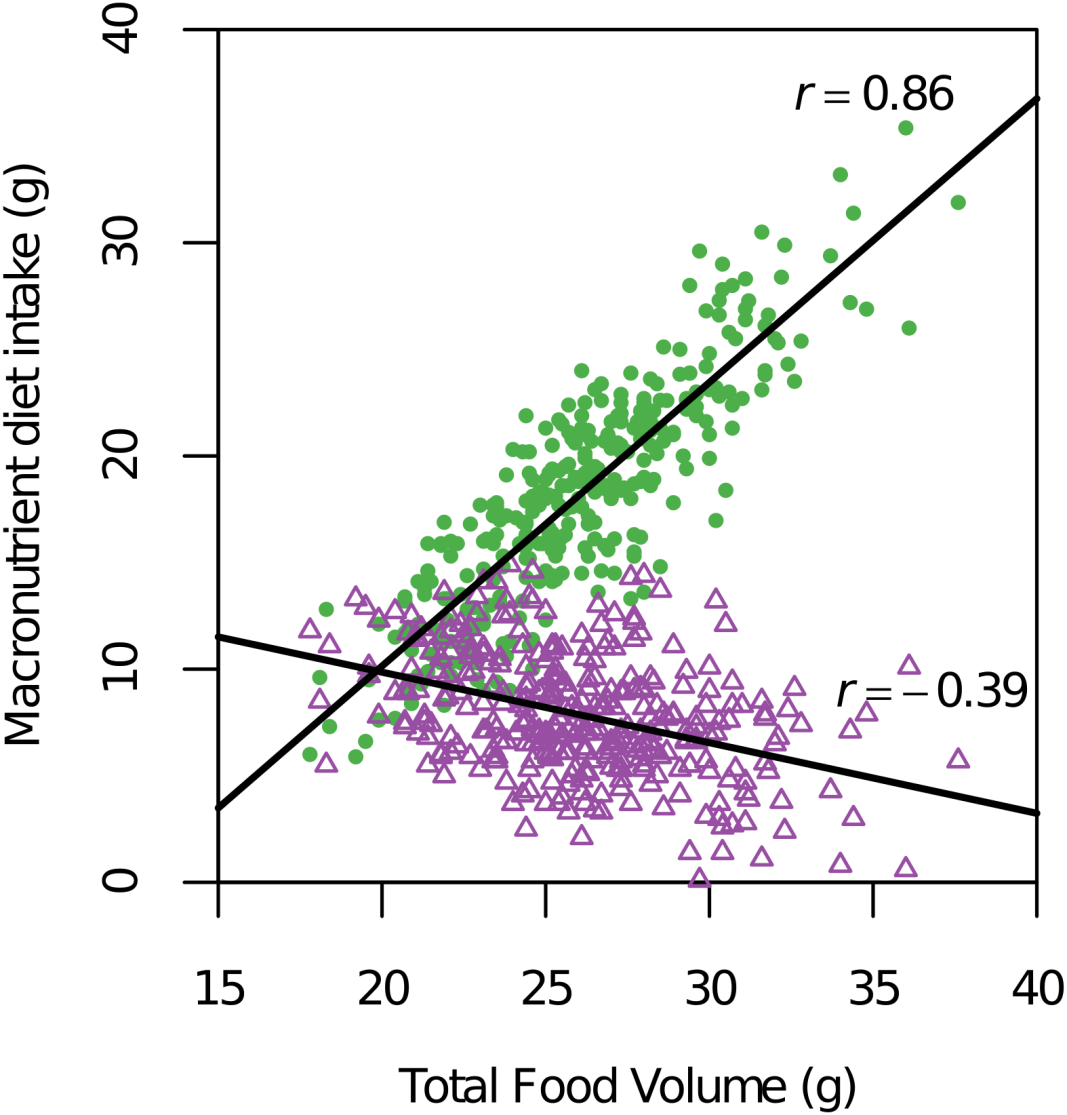
Preferential carbohydrate intake was positively correlated with total food volume in recombinant subcongenic-derived F_2_ mice. Legend: The macronutrient diet selection phenotype of preferential carbohydrate- versus fat-specific intake, unique to this genetic model, is illustrated. The total volume of food consumed in g was strongly and positively correlated with carbohydrate/protein (C/P) intake (*r* = 0.86, *P*<0.0001), but inversely correlated with fat/protein (F/P) intake (*r* = −0.39, *P*<.0001) in this macronutrient diet choice paradigm.

The intersecting pattern of QTLs determining the consumption of carbohydrate, total energy, and total food volume in this model was reflected by the inter-correlations among phenotypes. For example, the measures of total food consumed by recombinant F_2_ in both calories and g, showed a strong positive correlation (*r* = 0.85, *P*<0.0001; Table S2; Table S3). The lack of a perfect correlation between these two phenotypes can be attributed to variation in energy densities per weight (g) that exists between the two diet formulations. With respect to macronutrient-specific intake, the total volume of food consumed in g was strongly and positively correlated with carbohydrate/protein g intake (*r* = 0.86, *P*<0.0001), but inversely correlated with fat/protein g intake (*r* = −0.36, *P*<0.0001), as illustrated in Figure 4.

### Identification of QTL for body weight and body composition

**Figure 5:**
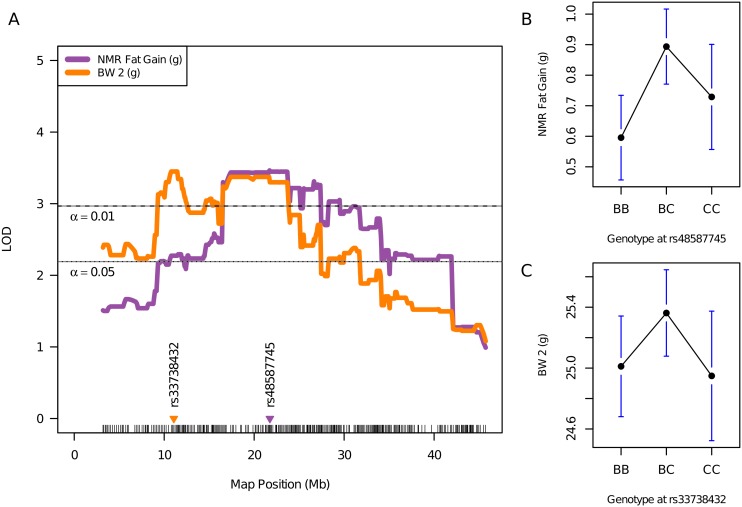
Short-term body fat and weight gain is controlled by a proximal chromosome 17 locus. Legend: Interval map of proximal chromosome 17 illustrates fine-mapped QTL for body fat gain and body weight gain after 10 d of macronutrient diet selection, in B6.CAST-17.1 subcongenic-derived F_2_ mice (left), and includes uncorrected phenotypic means for the peak marker of each QTL (right). The significant LOD threshold values for α = 0.01 and α = 0.05 were 2.97 and 2.19, respectively, as determined by 1,000 permutation tests. The locations of markers nearest the peak LOD scores are shown on the x-axis, e.g., *rs48587745*.

Following 10 d of macronutrient diet selection, two novel QTL not detected in our original F_2_ mapping study [8] were identified for BW2 (*rs33738432*, 11.06 Mb; LOD = 3.45) and body fat gain (*rs48587745*, 21.74 Mb; LOD = 3.46) (Table 2; Figure 5). It should be noted that the BW2 QTL is essentially the same as body weight gain over the 10 d period because BW1 was included as an additive covariate in the statistical model. A QTL influencing short-term body weight gain is located near marker *rs33738432*, with a CI between 3.19 and 31.84 Mb that partially overlaps the CI for a body fat gain QTL. A suggestive QTL for NMR2 lean mass also was identified (*rs51849267*, 31.44 Mb; LOD = 2.48). A significant QTL for epididymal fat pad weight was located at 21.31 Mb (*rs51776929*; LOD = 3.08), which replicated a previously identified *cast*-linked locus [39]. The effect of the CAST allele for each of these QTL was to increase body weight and body fat, relative to the B6 allele (Table 2). The short-term weight gain in recombinant F_2_ mice was positively correlated with total kcal consumption (*r* = 0.51, *P*<0.0001) and fat kcal (*r* = 0.22, *P*>0.01), but not carbohydrate kcal (*r* = 0.12, *P* = NS) (Table S3). Thus total energy-, rather than macronutrient-specific intake, may be the major contributor to short-term weight gain in this subcongenic-derived F_2_ population.

### Phenotypic analysis of subcongenic HQ17IIa mice

To refine the critical segment containing the *Mnic/Kcal2/Tfv1* QTLs, we evaluated the macronutrient selection phenotypes of an existing HQ17IIa subcongenic strain, harboring a 22.8 Mb segment of CAST genomic DNA on proximal MMU17 that is smaller than the B6.CAST-17.1 CAST donor segment (Figure 1). The time-course of the genotype effect on carbohydrate *vs.* fat intake in HQ17IIa mice over the 10 d period is shown in Figure S1. Compared to wild type (WT) controls, the HQ17IIa strain (C/C) selected/consumed similar, cumulative amounts of carbohydrate/protein kcal (73±7 C/C vs. 77±9 WT), fat/protein kcal (71±8 C/C vs. 65±8 WT), total kilocalories (127±2 CC vs. 129±3 WT), or total grams of food (27.8 C/C±0.7 vs. 29.1±1.2 WT) over the 10 d period. In addition, there were no strain differences in baseline body weight (35.5±0.5 g C/C vs. 36.8±0.7 g WT; *P* = 0.14) or percent lean mass (77±0.4% C/C vs. 76±0.6% WT; *P* = 0.16) (Table S6), therefore adjusting the animals’ food intake values for body size or metabolically active lean mass was deemed unnecessary. The phenotypes of HQ17IIa animals were determined in two experiments, including both a pilot study (unpublished) and a replicate study of 14 subcongenic and 14 controls (Figure S1). The results demonstrated clearly that HQ17IIa mice, compared to the appropriate B6 background controls, failed to exhibit the food intake phenotypes in question (preferential carbohydrate intake, increased total calorie/g intake), making it extremely likely that the causal genetic variant is located distal to the 26.08 Mb breakpoint in the mutant. Based on this result, the responsible region therefore can be reduced to a 19.044035 Mb interval located between 26.081867 and 45.125902 Mb (Figure 1), containing approximately 599 RefSeq genes.

### Analysis of sequence variation between strains

Using a combination of high-resolution fine-mapping and phenotypic analyses of subcongenic mouse strain HQ17IIa, we determined that the critical QTL spans the interval between 26.08 and 45.12 Mb (Figure 1). Based on the assumption that causative polymorphisms must be contained within the QTL region, we compiled a list of coding variants between CAST and B6 within these boundaries using data from the Wellcome Trust Sanger Institute Mouse Genome Project and the Mouse Phenome Database (http://phenome.jax.org/SNP). Thus we calculated, within the QTL boundaries, a total of 296 nonsynonymous coding SNPs in 71 genes. These genetic variants included nucleotide changes that result in the gain or loss of 3 stop codons, as well as 27 deletions and 19 insertions, e.g., some as long as 30 nucleotides; 5 inversions; 1 frame-shift variant, and 2 copy number variants. To look for variation that could predict altered protein function, missense mutations in the QTL intervals were evaluated by *in silico* analysis using the SIFT program [40]. SIFT predicts deleterious and tolerated substitutions for nonsynonymous coding (Cn) SNP based on the evolutionary conservation of amino acids within protein families. The results of this analysis predicted with high confidence that 14 of the 296 Cn SNP identified within the reduced QTL boundaries would have a possible detrimental effect. Although this program generates approximations, it can identify missense mutations that have potential functional significance and thus may warrant further investigation.

### Differential gene expression in *cast/cast* and *b6/b6* subcongenic-derived F_2_ mice

Gene expression analysis, independent of sequence-based analysis, can be helpful in identifying genes that are affecting a trait [24]. To highlight potentially relevant genes in the critical interval, a SAGE-seq experiment was performed to analyze gene expression in the hypothalamus of non-recombinant F_2_ progeny possessing *cast*/*cast* vs. *b6/b6* alleles across the subcongenic segment, and *b6/b6* alleles in the rest of the genome. Expression of these genes is regulated in *cis*, suggesting that local sequence variation within the QTL region accounts for the observed expression differences. The hypothalamus was chosen for analysis because it is an integration site for acute and long-term metabolic signals and is the tissue most likely to influence behavioral feeding and macronutrient-specific appetites [32]. Results from the SAGE-Seq analysis indicated that 55 genes or 9% of RefSeq genes in the 19.0 Mb interval were differentially expressed (DE) in the hypothalamus of littermate, non-recombinant subcongenic F_2_ mice (Table 3), selected from the 25% tails of the phenotype distribution for self-selected 10 d carbohydrate intake (see Methods). Gene expression was significantly increased (*cast/cast* genotype relative to *b6/b6*) in 13 genes and decreased in 42 genes (Table 3) in animals fed the macronutrient selection diet. Of the total 55 DE genes, *Glo1*, *Pla2g7* and *Pde9a* were validated previously in chow-fed B6.CAST-17.1*-(D17Mit19*-*D17Mit50)* subcongenic mice [20]. Because the differential expression of these three genes, by genotype, was independent of diet, they are unlikely to be causal for the phenotypic effects of *Mnic1/Kcal2/Tfv1* on macronutrient-specific intake.

**Table 3:** Differentially expressed genes in the hypothalamus of homozygous *cast/cast* vs. *b6/b6* subcongenic-derived F_2_ mice, located within the fine mapped QTL interval of 26.08–45.12 Mb.

Gene Symbol	Mean BB	Mean CC	Fold change	*P* value	Location (Mb)
Decr2	183.6	84.4	−2.174	0.0097	26.081211
Kifc5b	24.3	4.3	−5.682	≤0.0001	26.917091
Phf1	305.9	14.5	−21.075	≤0.0001	26.933127
Lemd2	36.6	13.2	−2.782	0.0001	27.189601
Gm10505	16.7	0.4	−47.588	≤0.0001	27.342460
Nudt3	906.9	417.9	−2.170	0.0057	27.579382
Uhrf1bp1	89.2	46.7	−1.911	0.0016	27.856490
Ppard	25.3	7.5	−3.352	0.0001	28.232754
Brpf3	732.6	49.8	−14.700	≤0.0001	28.801090
4930539E08Rik	106.1	368.2	3.470	≤0.0001	28.896397
Mtch1	3760.5	2032.3	−1.850	0.0009	29.332072
Tbc1d22b	29.7	12.0	−2.479	0.0015	29.549788
Gm6402	13.2	68.7	5.219	≤0.0001	30.394825
Glo1	1232.3	2575.0	2.090	≤0.0001	30.592866
Umodl1	14.7	1.1	−13.062	≤0.0001	30.954683
Rsph1	42.1	248.3	5.894	≤0.0001	31.255019
Pde9a	287.9	164.1	−1.754	0.0023	31.386234
Cbs	112.5	50.3	−2.236	0.0031	31.612623
Hsf2bp	16.6	59.6	3.584	≤0.0001	31.944769
Cyp4f16	52.9	4.5	−11.747	≤0.0001	32.536558
Zfp811	357.3	212.2	−1.684	0.0015	32.797406
Cyp4f14	44.6	3.0	−14.938	≤0.0001	32.905071
Zfp472	66.2	26.4	−2.511	≤0.0001	32.965814
Zfp763	80.9	23.9	−3.377	≤0.0001	33.016863
Morc2b	6.6	16.6	2.532	0.0048	33.135588
Zfp955a	638.3	256.4	−2.489	≤0.0001	33.241519
BC051226	32.1	10.8	−2.976	≤0.0001	33.908182
Zbtb22	262.4	15.6	−16.833	≤0.0001	33.915904
Wdr46	270.3	11.3	−24.002	≤0.0001	33.940660
Vps52	237.0	366.9	1.548	0.0099	33.955812
H2-K2	63.9	3.5	−18.311	≤0.0001	33.974786
AA388235	54.9	0.2	−348.820	≤0.0001	33.981492
H2-K1	129.8	57.6	−2.255	0.0030	33.996017
Ring1	1652.2	2534.3	1.534	0.0059	34.020792
Rxrb	969.4	208.9	−4.641	≤0.0001	34.031812
H2-DMa	47.4	107.1	2.258	0.0020	34.135182
Psmb9	11.8	29.0	2.463	0.0008	34.181988
Psmb8	66.4	8.8	−7.523	≤0.0001	34.197721
H2-Aa	125.9	1.2	−105.436	≤0.0001	34.282744
H2-Eb2	10.1	22.0	2.169	0.0093	34.325665
Agpat1	342.3	196.9	−1.739	0.0008	34.604262
Tnxb	147.2	48.2	−3.055	0.0002	34.670535
Neu1	310.1	151.3	−2.049	≤0.0001	34.931253
1110038B12Rik	179.2	5.6	−32.090	≤0.0001	34.950238
H2-Q1	677.6	390.8	−1.734	0.0014	35.320405
H2-Q4	26.9	1.3	−20.938	≤0.0001	35.379617
Ddr1	541.3	22.8	−23.761	≤0.0001	35.681567
Abcf1	1238.6	174.7	−7.089	≤0.0001	35.956819
A930015D03Rik	13.0	63.5	4.890	≤0.0001	35.994453
Rpp21	127.1	5.7	−22.294	≤0.0001	36.255645
Znrd1	448.4	5.3	−84.073	≤0.0001	36.954358
E130008D07Rik	34.4	2.6	−13.293	≤0.0001	43.146041
Pla2g7	773.3	6.2	−125.196	≤0.0001	43.568098
Cyp39a1	23.0	10.2	−2.252	0.0038	43.667425
Enpp4	323.1	534.0	1.653	0.0034	44.096308

Positive fold change indicates increased expression, negative value indicates decreased expression in homozygous *cast/cast* (CC) subcongenic-derived F_2_ mice (n = 12) relative to *b6/b6* (BB) subcongenic-derived F_2_ (n = 12). Genes were selected based on a significance value of *P*<0.01 and an expression change of ≥1.5-fold, based on sequence tag counts.

**Figure 6:**
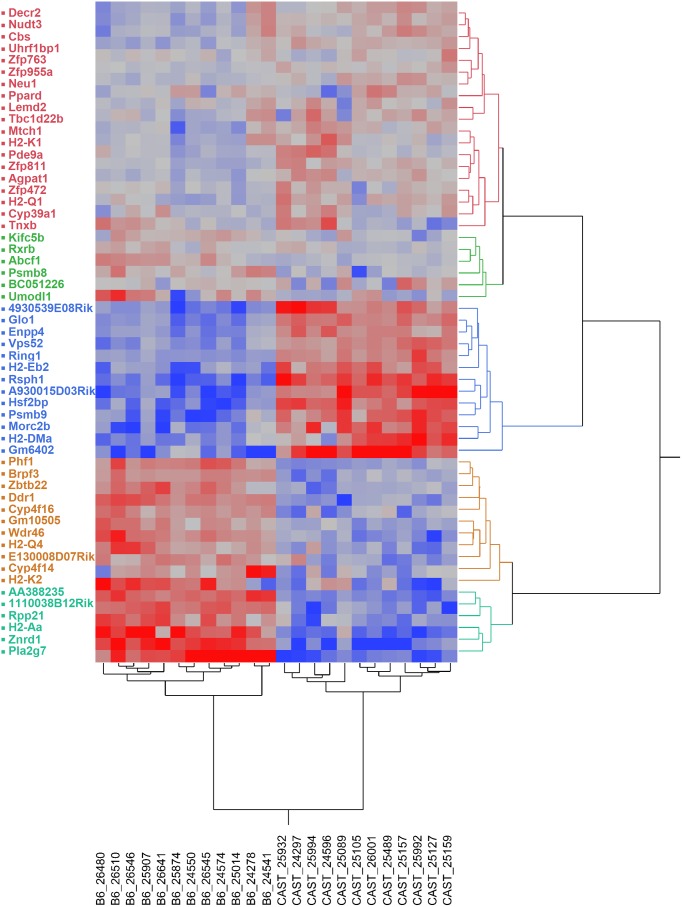
Hierarchical clustering of differential gene expression in the hypothalamus of non-recombinant B6.CAST-17.1-derived F_2_ mice. Legend: Gene expression was measured in hypothalamus on day 2 of macronutrient selection diet using tag-based transcriptome sequencing (n = 12 individual libraries per genotype). Animals used in this experiment were either *cast/cast* (carbohydrate-preferring) or *b6/b6* (fat-preferring) within the subcongenic segment, and *b6/b6* across the rest of the genome. Genes were filtered based on ≥1.5-fold change and *P* value≤0.01 using the DESeq method. The resulting 55 differentially expressed genes are displayed on the vertical axis; individual animals are clustered by genotype on the horizontal axis. Genes whose expression changed significantly in animals with the *cast/cast* genotype (higher carbohydrate consumption), relative to *b6/b6* (higher fat consumption), cluster together as increased (red) or decreased (blue) cells, with gray representing no change. Brighter shades of red and blue reflect higher degrees of up- and down-regulation.

Hierarchical clustering analyses of significant genes are illustrated in Figure 6, which revealed five main clusters of differentially regulated genes that were evident when the magnitude and direction of changes were considered. Genes whose expression changed significantly (*adjP*<0.01) in animals with the *cast/cast* genotype (higher carbohydrate consumption), relative to *b6/b6* (higher fat consumption), cluster together as increased (red) or decreased (blue) cells. Additional clusters with smaller magnitude of differences in gene expression level (less intense red or blue color), by genotype, are also shown.

WebGestalt was used to organize the differentially expressed genes into Gene Ontology (GO) Slim categories (Figure S3). Nearly 50% of these DE genes were classified as metabolism genes (Table S3), of which seven were associated with lipids and/or fatty acids (*Decr2*, *Ppard*, *Agpat1*, *Tnxb*, *Neu1*, *Pla2g7*, *Cyp39a1*; all down-regulated), and two with carbohydrate metabolism (*Glo1*, *Neu1*; up-regulated). We then used WebGestalt to visualize significantly enriched GO categories according to biological process, molecular function, and cellular component (Figure S4). The most significantly enriched GO category under biological process was antigen processing and presentation (7 genes, *adjP*<0.0001; Figure S4). Approximately 36% of the 55 differentially expressed genes have molecular functions related to metal ion binding (*adjP* = 0.0001). These include several transcription factors, e.g., *Ring1*, and members of the cytochrome P450 family. Long-chain fatty acid binding, represented by *Ppard* and *Cyp4f14*, was also noted (*adjP* = 0.004).

### Identification of potentially relevant candidate genes

To focus attention on candidate genes within the fine mapped *Mnic1/Kcal2/Tfv1* QTL interval, we employed both bio-informatic and genomic methods. We analyzed available mouse sequence data via http://phenome.jax.org/SNP, that was based on the following sources: GRCm38 coordinates, dbSNP 138 annotation, and Sanger data updates (28 Aug 2013). We identified 296 protein coding differences contained within the fine mapped interval. Next, using SIFT analysis, we determined *in silico* that 14 of these protein coding changes between strains are likely to be detrimental by impairing protein function. Strain-specific polymorphisms could also affect expression level, which can be valuable in identifying candidate genes [41]. To further understand the molecular mechanisms underlying *Mnic1/Kcal2/Tfv1*, hypothalamic gene expression was evaluated between homozygous *cast/cast* subcongenic and *b6/b6* wild-type F_2_ littermates using SAGE seq. We identified 55 differentially expressed positional candidates located within the critical 19.0 Mb region (Table 3) and among these, only two genes showed evidence of both altered mRNA abundance and a coding nonsynonymous SNP predicted as damaging: *Zfp811* and *Morc2b* (microrchidia 2B), including a stop gain codon in *Zfp811*. Available sequence data for *Zfp870*, while not differentially expressed in hypothalamus, shows seven nonsynonymous SNPs between B6 and CAST, two of which are calculated as damaging and one that predicts the gain of a premature stop codon. *Btnl6 (*butyrophilin-like 6) has two potentially deleterious SNPs that are predicted to result in the loss of a stop codon. The four candidate genes described above are located from ∼32.80 to 34.50 Mb, in close proximity to the *Mnic1* peak for preferential carbohydrate intake. However, nothing is known thus far about a functional role of these candidate genes in any food intake phenotypes.

To further search for possible candidates, we used the MGI ontologies database version 5.15 (www.informatics.jax.org) to examine available annotation for genes located in the minimal QTL region. Using the query terms food intake or energy intake, this search found only six biologically relevant genes: *Glp1r*, *Clps*, *Itpr3, Mut*, *Rcan2* and *Cdkn1a*. Based on our previous investigations, *Glp1r* remains a viable candidate gene for *Mnic1/Kcal2/Tfv1*, while evidence for *Clps* and *Itpr3* is lacking (see below and Discussion). Using bioinformatic resources, we found no evidence for an amino acid change or altered gene expression to support *Mut*, and *Rcan2* as candidates, but SIFT analysis predicted a deleterious SNP in *Cdkn1a* between the CAST and B6 strains. Our SAGE-Seq results showed clearly that *Cdkn1* is expressed in the hypothalamus, but was not differential between strains. We are unaware of any evidence for a physiological role of *Cdkn1a* in food intake regulation. A literature search uncovered one report suggesting that *Cdkn1a,* a cyclin-dependent kinase inhibitor, may have a role in the obesity-inducing effects of high-fat diet feeding beyond one generation [42]. However this study examined the effects of *Cdkn1a* activation to promote lipid accumulation, and did not explore its association with food intake or selection.

### Candidate gene analysis of *Itpr3*

Based on its physical location and possible physiological relevance to ingestive behavior phenotypes, we carried out candidate gene analyses of *Itpr3*. *Itpr3,* encoding inositol 1,4,5-triphosphate receptor, type 3, is a component of the taste transduction cascade and is located under the LOD peak for *Kcal2* at ∼27.1 Mb. Specifically, the intracellular calcium channel ITPR3 releases calcium from the endoplasmic reticulum in response to signals from small molecules and proteins including nucleotides, kinases, and phosphatases, as well as non-enzyme proteins [43], [44]. The ITPR3 is widely expressed in tissues including taste buds, olfactory epithelium, brain, epidermis, intestine, kidney, pancreatic islets and stomach [45], [46]. It has been suggested that *Itpr3* may play a role in macronutrient diet choice [47]. We therefore investigated the possibility that *Itpr3* contributes to the effects of *Mnic1/Kcal2*. We examined the expression of *Itpr3* in the hypothalamus of B6.CAST-17.1 subcongenic compared to wild-type mice using quantitative PCR, but found no difference in expression level. Next, using allele-specific PCR, we verified a non-synonymous coding SNP in *Itpr3* that changes G to T at 27,098,333 base pair (bp) of chromosome 17 and produces a alanine-to-serine missense mutation at position 821 (A821S). The amino acid change resulting from this polymorphism was classified as ‘tolerated’ by SIFT [40]. Next, we hypothesized that altered taste perception of the experimental diets might contribute to carbohydrate preference in our selection paradigm. Thus we examined the taste preferences of B6.CAST-17.1 subcongenic mice for sucrose using 48-h two-bottle tests. The result was that B6.CAST-17.1 displayed normal behavioral responses to sucrose compared to wild type controls (Figure S2). Although the strain difference in ITPR3 protein sequence could possibly affect protein function, it did not severely impair sensitivity for sweet stimuli in our genetic model, as has been reported in ITPR3 knockout mice [46]. Therefore the phenotype of preferential carbohydrate intake does not appear to be driven by a preference for sweet taste, and must depend on some other mechanism. Nonetheless, the location of *Itpr3* coincides closely with the peak map position for *Kcal2/Tfv1* (Table 2), allowing the possibility that *Itpr3* contributes to total calorie intake and/or food volume, but this has not been determined.

## Discussion

In this study, we have clearly confirmed and refined the locations of QTL regulating self-selected macronutrient intake-carbohydrate (*Mnic1*), as well as the consumption of total kilocalories (*Kcal2*) and total food volume (*Tfv1*). These food intake QTL were originally identified through genome-wide mapping [8], [23] and now have been reproduced in a subcongenic-derived F_2_ mapping population. The absence of genetic linkage for self-selected fat intake in the results underscores the carbohydrate-specific effect of this mouse chromosome 17 locus on macronutrient selection. The fine mapped *Mnic1/Kcal2/Tfv2* QTL was then further de-limited by evaluating macronutrient diet selection phenotypes in an interval-specific subcongenic strain. Consequently, we are now able to infer that a 19.04 Mb interval between 26.08 and 45.12 Mb harbors the responsible sequence variation regulating these food intake traits.

To our knowledge, no other genetic loci for macronutrient preference have been reported in laboratory rodents until recently. Specifically, a number of food consumption QTL in animals selecting their intake from separate macronutrient (protein, carbohydrate and fat) sources were mapped in a LOU/C×Fischer 344 F_2_ rat population [48]. Two QTL influencing the percentage of carbohydrate consumed were identified on rat chromosomes 10 (*Foco17*) and 20 (*Foco23*). These rat QTL regions share synteny blocks with the fine mapped mouse *Mnic1* region for preferential carbohydrate intake described in the current study (www.rgd.mcw.edu), providing further support for its authenticity.

Our study also revealed two partially overlapping QTL, affecting short term gains in both body fat (NMR fat gain) and body weight (BW 2) (Figure 5) (Table 2), positioned between the centromere and lower boundary of the 95% CI for *Mnic1* (preferential carbohydrate intake QTL). The body weight/fat gain QTL peaks are situated ∼10–20 Mb away from the food intake QTL (Figures 3 & 5). The separation of these energy balance loci, *Mnic1/Kcal2/Tfv1* for food intake and the newly discovered locus regulating short term body weight/fat gain, suggests the possibility of different quantitative trait genes for two energy balance traits, i.e., intake and storage. This region co-localizes with at least four other obesity-related QTLs, previously mapped to this region in other strains [49]–[51], including the *Obrq13* locus where the A/J-derived allele protects against the diet-induced obesity that characterizes B6 males when fed a high-fat diet [37]. In the current study, the QTL for body fat gain after 10 d of macronutrient diet selection coincides with a locus for retroperitoneal fat pad weight identified previously in a C57BL/6J×CAST/Ei intercross [39]. The effect of the *cast* allele on body fat gain, although modest, was highly significant (*P*<0.001; Table 2) and occurred over a very short period of time. This finding is remarkable because the CAST strain has been characterized for obesity-resistant phenotypes [52], [53] in studies employing a single, obesity-inducing diet of moderate fat content, while the *b6* allele usually confers obesity in mapping crosses [37], [51].

Among the genes that exhibited differential mRNA expression between *cast/cast* and *b6/b6* in the hypothalamus, three showed ontologies with possible relevance to eating behavior phenotypes. Specifically, *Decr2*, *Ppard*, and *Agpat1* are associated with fatty acid oxidation or lipid metabolism and were down-regulated in *cast/cast* subcongenic F_2_ compared to *b6/b6*. *Ppard* (peroxisome proliferator-activated receptor, delta), also known as *Nr1c2*, is a lipid-activated transcription factor that forms an obligate heterodimer with the retinoid × receptor to induce fatty acid transport and oxidation [54]. In our SAGE-Seq experiment (Table 3), *Ppard* and its binding partner *Rxrb* (retinoid × receptor beta) showed 3-fold and nearly 5-fold down-regulation, respectively, in the hypothalamus of carbohydrate- compared to fat-preferring mice. *Ppard* is the most highly expressed PPAR isoform in the CNS, where it is enriched in medio-basal hypothalamus [55], an area involved in energy homeostasis. Evidence for a role of *Ppard* in feeding behavior is limited, e.g., mice with neuron-specific deletion of *Ppard* showed a blunted feeding response to fasting [56]. There are no reports of an association of *Decr2* or *Agpat1* with food intake regulation. Nevertheless, *Decr2* encodes 2,4-dienoyl CoA reductase, an enzyme which catalyzes a reaction considered to be the rate-limiting step in the beta-oxidation of polyunsaturated fatty acids [57]. It is intriguing that expression of a QTL region gene with a key metabolic function in lipid metabolism is significantly decreased in the hypothalamus of carbohydrate-preferring mice. More studies will be needed to address whether the decreased expression of any of these lipid-related genes is a cause or consequence of macronutrient diet selection behavior.

Three positional candidates previously investigated by our group (*Clps*, *Glo1,* and *Glp1r*) reside within the critical interval. Pancreatic colipase (*Clps*) at 28.5 Mb, encodes a cofactor involved in fat digestion; however, sequencing uncovered only two silent nucleotide polymorphisms [8] and no expression differences were observed in the current study. *Glo1* (glyoxalase I), located at 30.59 Mb, is involved in the detoxification of methylglyoxal, a highly reactive dicarbonyl compound formed as a by-product of glycolysis, which can be toxic to the genome and proteome [58]. A mechanistic link between *Glo1* and food preferences has not been established. The strain difference in hypothalamic *Glo1* expression, observed in the current study, replicates previous observations of an up-regulation of *Glo1* in B6.CAST-17 carbohydrate-preferring congenic mice compared to fat-preferring wild type mice [21], [22]. These observations initially led us to hypothesize that high carbohydrate consumption produces a higher flux of MG, leading to increased *Glo1* expression. However, our studies have consistently demonstrated a several-fold higher *Glo1* mRNA and enzyme activity in B6.CAST-17 congenic and subcongenic mice, whether animals were fed standard rodent chow, high- or low-carbohydrate diets, or macronutrient selection diets [20]–[22]. Together, these findings provide convincing evidence for intrinsic strain variation in *Glo1* expression, independent of the level of carbohydrate intake, and when combined with the lack of evidence for a functional SNP [21], weaken support for *Glo1* as a candidate for *Mnic1*. *Glp1r*, located nearby at ∼30.90 Mb under a sharp LOD curve inflection (Figure 3), remains a strong candidate for *Mnic1* (preferential carbohydrate intake) based on its physiological roles in glucose homeostasis and gastric emptying. We demonstrated previously that *Glp1r* is differentially expressed in both the pancreas and stomach (yet in opposite direction), but not in hypothalamus of B6.CAST-17 congenic mice compared to B6 [21]. Furthermore, *Glp1r* expression in the stomach of F1 heterozygous mice showed allelic imbalance, i.e., a significant deviation from the theoretical 50∶50 allelic ratio [23], suggesting that a stomach-specific, *cis*-acting regulatory element may underlie the strain difference in gastric *Glp1r* expression. Sequencing of *Glp1r* identified a non-synonymous coding SNP (C416Y) between strains which could have functional implications in the downstream regulation of protein activity [21]. These collective results, along with the close proximity of *Glp1r* near the refined *Mnic1* peak (32.49 Mb), support its further investigation and the consideration of a possible gastrointestinal mechanism.

We combined high-density SNP-mapping and subcongenic strain analyses to reduce the critical QTL interval for preferential carbohydrate and total food intake to a 19.0 Mb region on proximal Chr 17. We also obtained evidence that suggests the existence of separate QTLs determining food intake and short term body weight/fat gain and raises the prospect of distinct molecular genetic mechanisms for these energy balance phenotypes. To approach the question of which gene or genes within the fine-mapped 19.0 Mb interval could be causal, we examined available sequence data and gene ontologies, and performed comprehensive gene expression profiling in the hypothalamus of *cast/cast* and *b6/b6* non-recombinant F_2_ controls. From these analyses, genes such as *Zfp811*, *Zfp870*, and *Btnl6* were shown to be differentially expressed and also to contain stop codons, although they have no known biology related to food intake regulation. In particular, *Decr2*, *Ppard* and *Agpat1* drew our attention as appealing candidates because of their involvement in lipid metabolism and down-regulation in carbohydrate-preferring animals. Additional studies, e.g., genetic modifications targeting priority candidates to determine their phenotypic effects, are required to uncover their potential contributions to the effects of *Mnic1/Kcal2/Tfv1*.

## Supporting Information

Figure S1 **Title: HQ17IIa mice, compared to WT controls, did not exhibit increased intake of carbohydrate kcal, total kcal and total food volume.** Legend: Daily consumption of (A) carbohydrate/protein kcal (C/P) versus (B) fat/protein kcal (F/P), total kcal (C) and total food volume (D) in HQ17IIa (CC) and wild type (WT) mice. Values are mean ± SE. The HQ17IIa subcongenic mouse strain is homozygous for CAST/EiJ (CAST) alleles from 3.19 to 26.08 Mb on MMU 17 (Figure 1). These results show clearly that HQ17IIa mice, compared to the appropriate B6 background controls, failed to exhibit the food intake phenotypes in question, making it extremely likely that the causal genetic variant is located distal to the 26.08 Mb breakpoint in the mutant.
(TIF)

Figure S2 **Mean taste preference ratios of B6.CAST-17.1 WT and subcongenic mice using 48-hour two-bottle (tastant vs. distilled water) preference tests.** Legend: Behavioral responses to sucrose (15, 30, and 60 mM) in B6.CAST-17.1 WT and subcongenic mice. The values are means ± SE (n = 7 per strain group). #*P*<0.05, *P<0.001 vs. a preference ratio of 0.50, using the one-sample *t*-test.
(TIFF)

Figure S3 **Biological process categories.** Legend: WEB-based Gene SeT AnaLysis Toolkit (WebGestalt; http://bioinfo.vanderbilt.edu/webgestalt/) was used to organize the differentially expressed genes into Gene Ontology (GO) biological process categories. Nearly 50% of all 55 DE genes were classified as metabolism genes, of which seven were associated with lipids and/or fatty acids (*Decr2*, *Ppard*, *Agpat1*, *Tnxb*, *Neu1*, *Pla2g7*, *Cyp39a1*; all down-regulated), and two with carbohydrate metabolism (*Glo1*, *Neu1*; up-regulated).
(TIFF)

Figure S4 **Enriched gene ontology categories.** Legend: WebGestalt was also used to visualize significantly enriched GO categories according to biological process, molecular function, and cellular component. For biological process, the most significantly enriched GO category was antigen processing and presentation (7 genes, *adjP*<0.0001). Approximately 36% of the 55 differentially expressed genes have molecular functions related to metal ion binding (*adjP* = 0.0001), including several transcription factors, e.g., *Ring1*, and members of the cytochrome P450 family. Long-chain fatty acid binding, represented by *Ppard* and *Cyp4f14*, was also noted (*adjP* = 0.004).
(TIF)

Table S1 **SNP markers and their physical and genetic positions.** Legend: SNP markers and their chromosomal positions according to http://genome.ucsc.edu. Mm9 sequence was obtained from the Build 37 assembly by NCBI and the Mouse Genome Sequencing Consortium. Excluded, markers not used in the analysis.
(DOCX)

Table S2 **Correlation matrix for nutrient intake phenotypes and baseline, pre-diet selection characteristics in the recombinant congenic F_2_ population.** Legend: Carbohydrate/protein (C/P), fat/protein (F/P), total kilocalories (kcal) and total food volume (g) consumed; body weight (BW 1), body fat (NMR1 fat), and body lean mass (NMR1 lean) when diet selection began. ^a^ *P*<0.05; ^b^ *P*<0.01; ^c^ *P*<0.001, ^d^ *P*<0.0001.
(DOCX)

Table S3 **Correlation matrix for nutrient intake phenotypes and post-diet selection characteristics in the recombinant congenic F_2_ population.** Legend: Carbohydrate/protein (C/P), fat/protein (F/P), total kilocalories (kcal) and total food volume (g) consumed; body weight (BW 2), body weight gain from BW 1 to BW 2; body fat (NMR2 fat) and body lean mass (NMR2 lean) after 10 d macronutrient diet selection; fat mass gain (Fat gain 12) and lean mass gain (lean gain12) over 10 d diet selection. ^a^ *P*<0.05; ^b^ *P*<0.01; ^c^ *P*<0.001, ^d^ *P*<0.0001.
(DOCX)

Table S4 **Correlation matrix for nutrient intake phenotypes and baseline, pre-diet selection characteristics in the non-recombinant congenic F_2_ population.** Legend: Carbohydrate/protein (C/P), fat/protein (F/P), total kilocalories (kcal) and total food volume (g) consumed; body weight (BW 1), body fat (NMR1 fat), and body lean mass (NMR1 lean) when diet selection began. ^a^ *P*<0.05; ^b^ *P*<0.01; ^c^ *P*<0.001, ^d^ *P*<0.0001.
(DOCX)

Table S5 **Correlation matrix for nutrient intake phenotypes and post-diet selection characteristics in the non-recombinant congenic F_2_ population.** Legend: Carbohydrate/protein (C/P), fat/protein (F/P), total kilocalories (kcal) and total food volume (g) consumed; body weight (BW 2), body weight gain from BW 1 to BW 2; body fat (NMR2 fat) and body lean mass (NMR2 lean) after 10 d macronutrient diet selection; fat mass gain (Fat gain 12) and lean mass gain (lean gain12) over 10 d diet selection. ^a^ *P*<0.05; ^b^ *P*<0.01; ^c^ *P*<0.001, ^d^ *P*<0.0001.
(DOCX)

Table S6 **Body weight and composition data for HQ17IIa sub-congenic mice.** Legend: Values (mean ± SE) were obtained immediately before and then after the 10 d period of macronutrient diet selection.
(DOCX)
