# Supplementary figures and images for: High-Resolution Mapping of a Genetic Locus Regulating Preferential Carbohydrate Intake, Total Kilocalories, and Food Volume on Mouse Chromosome 17
Source: PLoS One. 2014 Oct 20;9(10):e110424. doi: 10.1371/journal.pone.0110424 (PMC4203797; doi:10.1371/journal.pone.0110424)

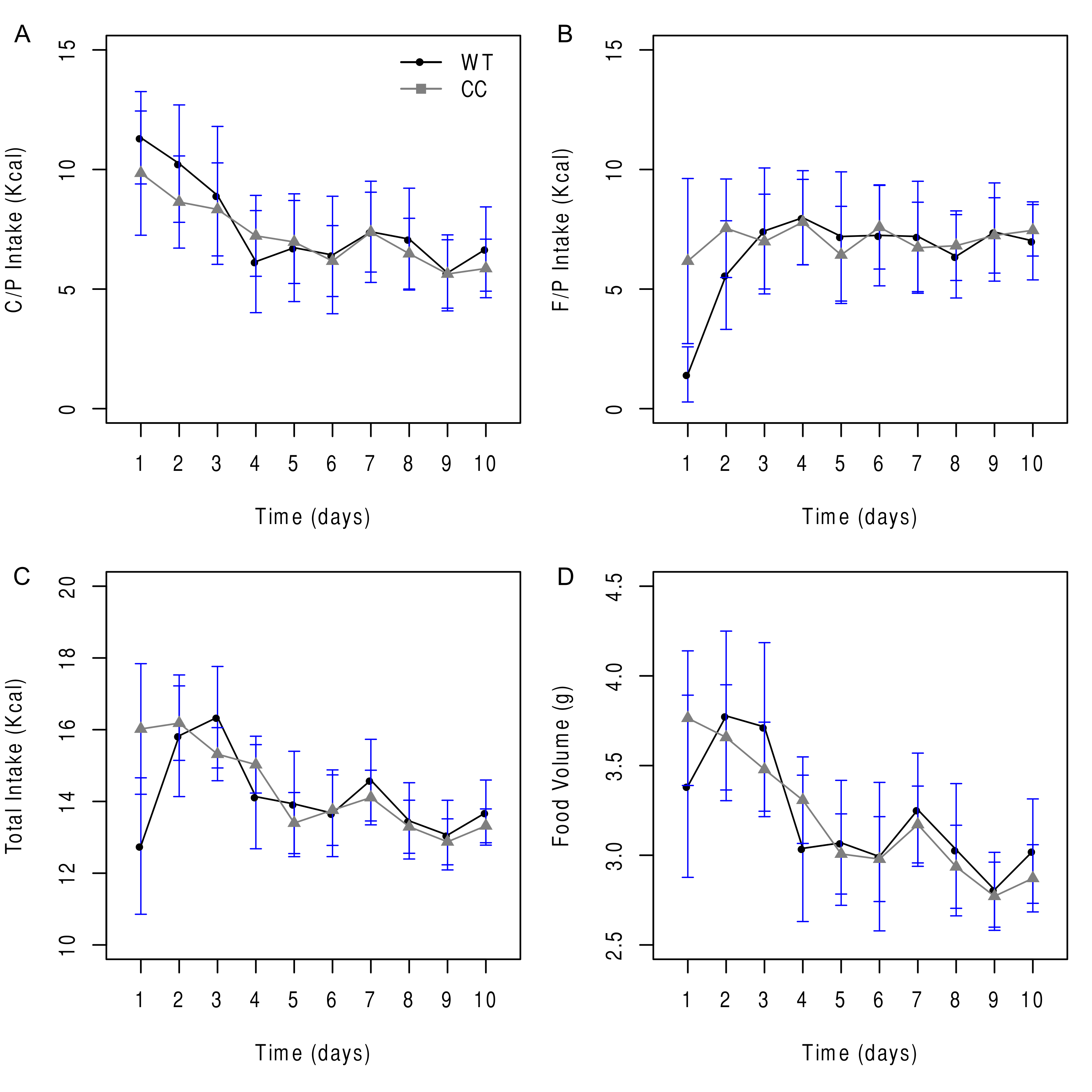

Supplement: Figure S1 — Title: HQ17IIa mice, compared to WT controls, did not exhibit increased intake of carbohydrate kcal, total kcal and total food volume. Legend: Daily consumption of (A) carbohydrate/protein kcal (C/P) versus (B) fat/protein kcal (F/P), total kcal (C) and total food volume (D) in HQ17IIa (CC) and wild type (WT) mice. Values are mean ± SE. The HQ17IIa subcongenic mouse strain is homozygous for CAST/EiJ (CAST) alleles from 3.19 to 26.08 Mb on MMU 17 (Figure 1). These results show clearly that HQ17IIa mice, compared to the appropriate B6 background controls, failed to exhibit the food intake phenotypes in question, making it extremely likely that the causal genetic variant is located distal to the 26.08 Mb breakpoint in the mutant. (TIF) [file pone.0110424.s001.tif]

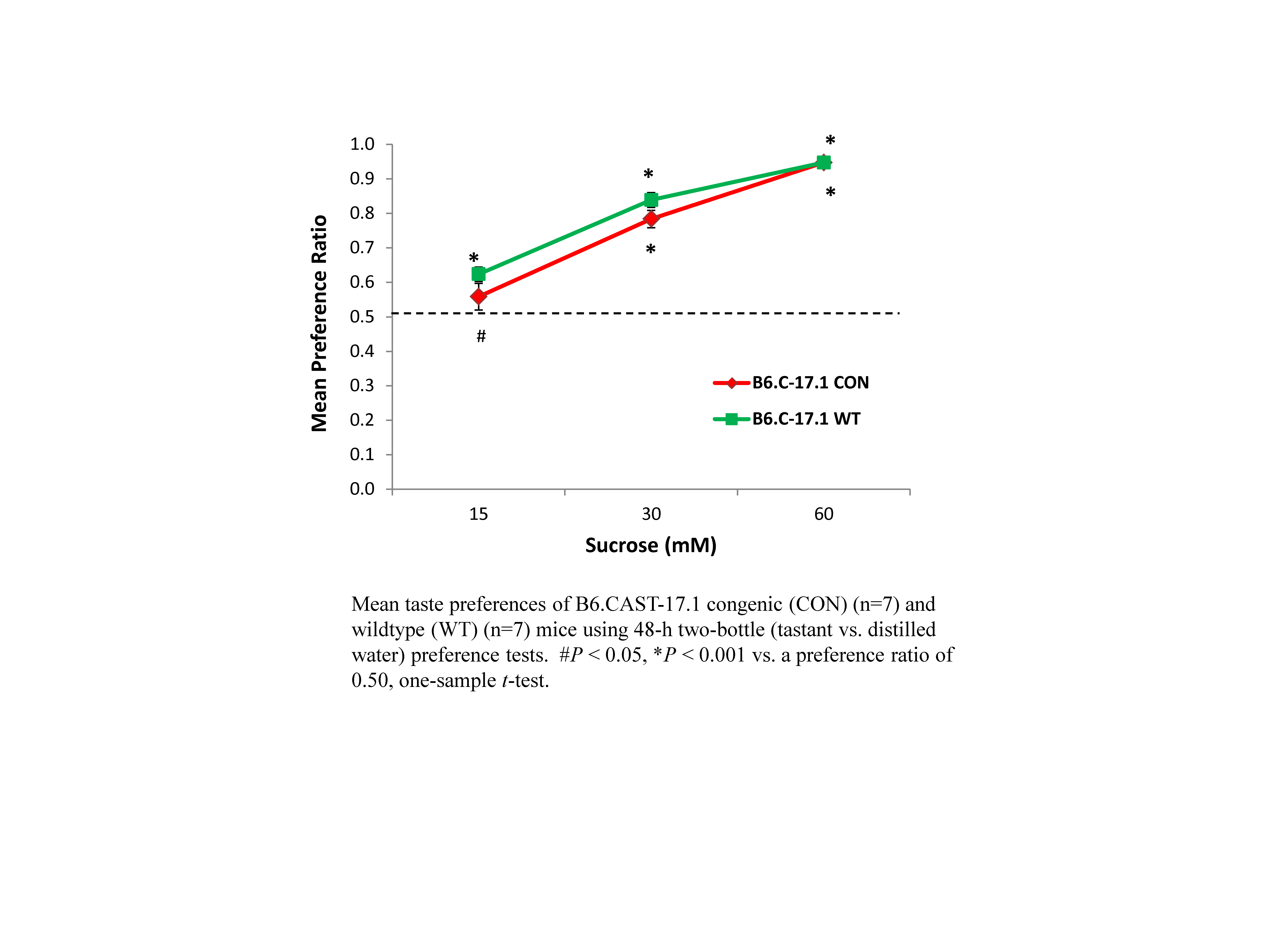

Supplement: Figure S2 — Mean taste preference ratios of B6.CAST-17.1 WT and subcongenic mice using 48-hour two-bottle (tastant vs. distilled water) preference tests. Legend: Behavioral responses to sucrose (15, 30, and 60 mM) in B6.CAST-17.1 WT and subcongenic mice. The values are means ± SE (n = 7 per strain group). #P<0.05, *P<0.001 vs. a preference ratio of 0.50, using the one-sample t-test. (TIFF) [file pone.0110424.s002.tiff]

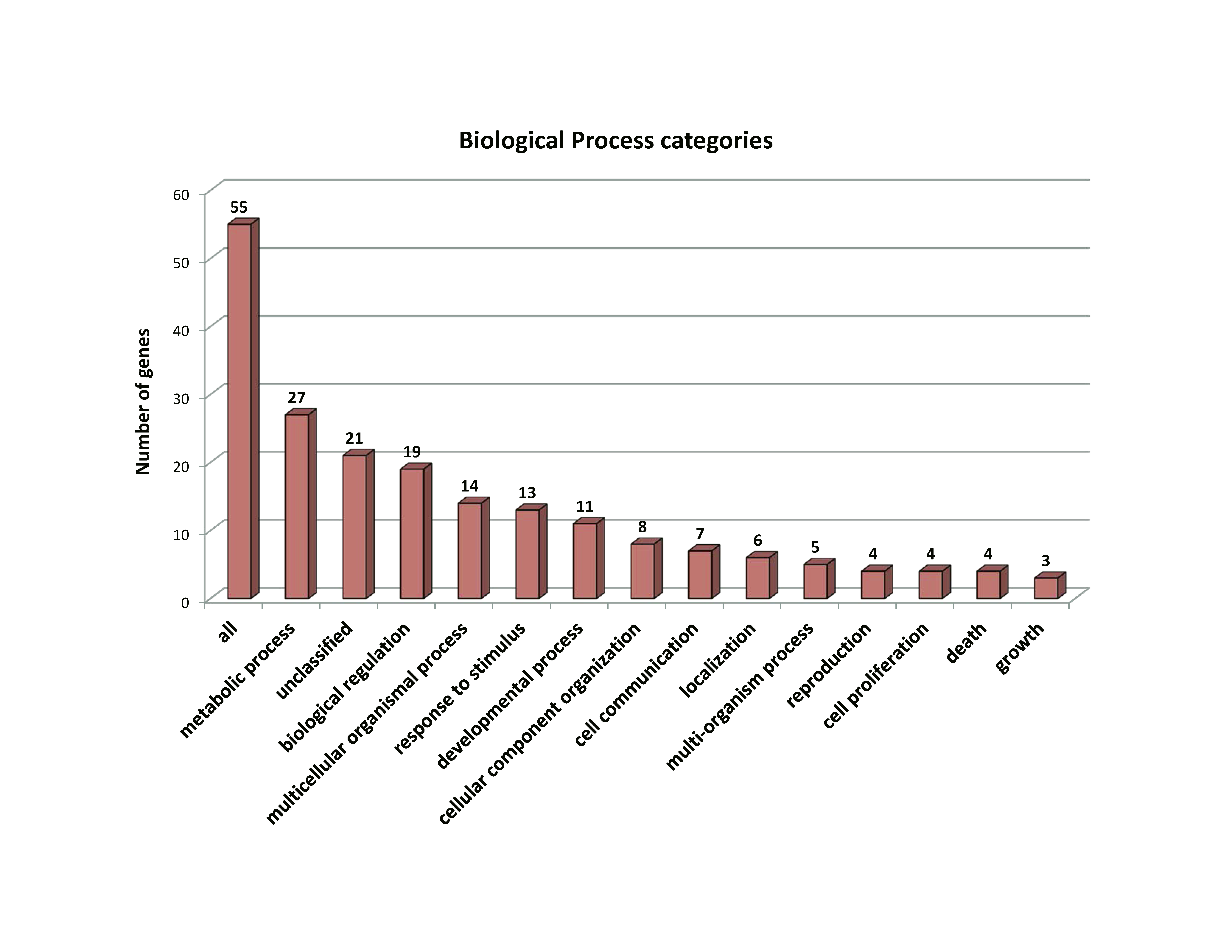

Supplement: Figure S3 — Biological process categories. Legend: WEB-based Gene SeT AnaLysis Toolkit (WebGestalt; http://bioinfo.vanderbilt.edu/webgestalt/) was used to organize the differentially expressed genes into Gene Ontology (GO) biological process categories. Nearly 50% of all 55 DE genes were classified as metabolism genes, of which seven were associated with lipids and/or fatty acids (Decr2, Ppard, Agpat1, Tnxb, Neu1, Pla2g7, Cyp39a1; all down-regulated), and two with carbohydrate metabolism (Glo1, Neu1; up-regulated). (TIFF) [file pone.0110424.s003.tiff]
