# Supplementary material for: High-Resolution Mapping of a Genetic Locus Regulating Preferential Carbohydrate Intake, Total Kilocalories, and Food Volume on Mouse Chromosome 17
Source: PLoS One. 2014 Oct 20;9(10):e110424. doi: 10.1371/journal.pone.0110424 (PMC4203797; doi:10.1371/journal.pone.0110424)
Supplement: Table S1 — SNP markers and their physical and genetic positions. Legend: SNP markers and their chromosomal positions according to http://genome.ucsc.edu. Mm9 sequence was obtained from the Build 37 assembly by NCBI and the Mouse Genome Sequencing Consortium. Excluded, markers not used in the analysis. (DOCX) [file pone.0110424.s005.docx]

Table 1. SNP markers and their physical and genetic positions

| Marker | Chromosome | mm9 (Mb) | cM |
| --- | --- | --- | --- |
| rs49640908 | 17 | 3.197604 | 3.197604 |
| rs49477544 | 17 | 3.290175 | 3.197604 |
| rs33665234 | 17 | 3.414322 | Excluded |
| rs33069377 | 17 | 3.477277 | 3.197604 |
| rs6153222 | 17 | 3.543171 | 3.197604 |
| rs33653408 | 17 | 3.679781 | 3.197604 |
| rs33651689 | 17 | 3.862814 | 3.385713 |
| rs13482843 | 17 | 4.044735 | 3.385713 |
| rs33642110 | 17 | 4.277046 | 3.385714 |
| rs33633320 | 17 | 4.493197 | 3.385714 |
| rs33683300 | 17 | 4.720948 | 3.385714 |
| rs33678427 | 17 | 4.887993 | Excluded |
| rs33680196 | 17 | 4.942684 | 3.385714 |
| rs33670622 | 17 | 5.132781 | 3.385714 |
| rs46392585 | 17 | 5.321347 | 3.385714 |
| rs46843561 | 17 | 5.418555 | 3.385714 |
| rs48030175 | 17 | 5.448507 | 3.385714 |
| rs51277965 | 17 | 5.726914 | 3.763920 |
| rs47725817 | 17 | 5.899118 | 3.763920 |
| rs50649694 | 17 | 6.009735 | 3.763920 |
| rs48813706 | 17 | 6.124125 | Excluded |
| rs52003095 | 17 | 6.252444 | Excluded |
| rs46748968 | 17 | 6.520249 | Excluded |
| rs33695293 | 17 | 6.943479 | 3.951728 |
| rs33857400 | 17 | 7.101391 | 4.139517 |
| rs33688800 | 17 | 7.290631 | 4.706434 |
| rs33684649 | 17 | 7.466559 | 4.706434 |
| rs33683849 | 17 | 7.524306 | Excluded |
| rs49126655 | 17 | 7.732951 | 4.706434 |
| rs45897981 | 17 | 7.931709 | 4.706434 |
| rs33516125 | 17 | 8.091584 | 4.706435 |
| rs33137358 | 17 | 8.122085 | 4.706435 |
| rs48687642 | 17 | 8.284598 | 4.706435 |
| rs48213049 | 17 | 8.368421 | 4.706435 |
| rs49056705 | 17 | 8.449561 | 4.706435 |
| rs51943262 | 17 | 8.535623 | Excluded |
| rs50569488 | 17 | 8.634728 | Excluded |
| rs51615552 | 17 | 8.813517 | 4.706435 |
| rs46857129 | 17 | 8.988855 | 4.706435 |
| rs50236138 | 17 | 9.167669 | 4.893933 |
| rs46011679 | 17 | 9.340772 | 4.893933 |
| rs51309079 | 17 | 9.514859 | 5.270353 |
| rs33716026 | 17 | 9.689944 | 5.270353 |
| rs33258859 | 17 | 9.861175 | 5.456211 |
| rs33167095 | 17 | 10.042912 | 5.456211 |
| rs33505770 | 17 | 10.225927 | 5.641353 |
| rs33696104 | 17 | 10.404972 | 6.200265 |
| rs51655217 | 17 | 10.547190 | Excluded |
| rs33750661 | 17 | 10.689450 | 6.200265 |
| rs33741613 | 17 | 10.832581 | Excluded |
| rs33744639 | 17 | 10.977385 | 6.571717 |
| rs33745159 | 17 | 11.119486 | 6.571717 |
| rs33738432 | 17 | 11.261578 | 6.571717 |
| rs33737976 | 17 | 11.403752 | 6.571717 |
| rs33728065 | 17 | 11.543650 | 6.571717 |
| rs33313589 | 17 | 11.644384 | 6.571717 |
| rs33157111 | 17 | 11.744532 | 6.571717 |
| rs33723709 | 17 | 11.828209 | 6.571718 |
| rs33715706 | 17 | 11.924715 | 6.571718 |
| rs47406766 | 17 | 12.060745 | 6.571718 |
| rs48679559 | 17 | 12.196968 | 6.756915 |
| rs33296434 | 17 | 12.292180 | 6.756915 |
| rs47392319 | 17 | 12.344559 | 6.756915 |
| rs47570998 | 17 | 12.447916 | Excluded |
| rs47127344 | 17 | 12.470948 | 6.756915 |
| rs50382495 | 17 | 12.517871 | 6.756916 |
| rs13464117 | 17 | 12.604389 | 6.756916 |
| rs51688353 | 17 | 12.650457 | Excluded |
| rs50096453 | 17 | 12.792070 | 7.129384 |
| rs16810039 | 17 | 12.843497 | 7.129384 |
| rs47710754 | 17 | 12.868265 | 7.315441 |
| rs49880468 | 17 | 12.904924 | Excluded |
| rs47160713 | 17 | 13.070694 | 7.315441 |
| rs51403187 | 17 | 13.136253 | Excluded |
| rs50554014 | 17 | 13.209287 | 7.315441 |
| rs51828280 | 17 | 13.397438 | 7.315441 |
| rs47353483 | 17 | 13.419735 | Excluded |
| rs46025507 | 17 | 13.548603 | 7.315441 |
| rs49203520 | 17 | 13.719083 | Excluded |
| rs47937429 | 17 | 13.853766 | 7.315441 |
| rs47798477 | 17 | 13.987809 | 7.315441 |
| rs48488829 | 17 | 14.042248 | 7.315441 |
| rs51820165 | 17 | 14.183227 | 7.315441 |
| rs13482885 | 17 | 14.336401 | 7.315441 |
| rs47875424 | 17 | 14.481351 | 7.315442 |
| rs46515655 | 17 | 14.536576 | 7.315442 |
| rs51801646 | 17 | 14.613579 | 7.315442 |
| rs47627120 | 17 | 14.706144 | Excluded |
| rs47223713 | 17 | 14.807817 | 7.502290 |
| rs47094196 | 17 | 14.840101 | 7.502290 |
| rs50942228 | 17 | 14.966534 | 7.502291 |
| rs51959766 | 17 | 15.052707 | 7.502291 |
| rs50208445 | 17 | 15.198034 | Excluded |
| rs49853892 | 17 | 15.370911 | 7.689141 |
| rs50596095 | 17 | 15.486225 | 7.875990 |
| rs47501797 | 17 | 15.510531 | 7.875990 |
| rs51382473 | 17 | 15.538848 | 7.875991 |
| rs13464868 | 17 | 15.613143 | 7.875991 |
| rs48970296 | 17 | 15.692566 | 7.875991 |
| rs46172621 | 17 | 15.862241 | 8.062871 |
| rs51508219 | 17 | 15.943366 | 8.062871 |
| rs49009610 | 17 | 16.067791 | 8.062871 |
| rs6343309 | 17 | 16.188597 | 8.437841 |
| rs51160774 | 17 | 16.311087 | 8.437842 |
| rs33754019 | 17 | 16.437377 | 8.437842 |
| rs33750505 | 17 | 16.556153 | 8.437842 |
| rs33747676 | 17 | 16.674276 | 9.192459 |
| rs33744973 | 17 | 16.796914 | 9.192459 |
| rs33750473 | 17 | 16.919611 | 9.192459 |
| rs33745888 | 17 | 17.026311 | 9.823893 |
| rs50685827 | 17 | 17.160622 | Excluded |
| rs46167454 | 17 | 17.363257 | Excluded |
| rs13459386 | 17 | 17.530896 | 10.645046 |
| rs33759383 | 17 | 17.700596 | 10.645046 |
| rs33757544 | 17 | 17.850413 | 10.645047 |
| rs16798559 | 17 | 17.980945 | Excluded |
| rs50390299 | 17 | 18.107457 | 10.645047 |
| rs50412415 | 17 | 18.197659 | Excluded |
| rs48901700 | 17 | 18.288389 | Excluded |
| rs46329114 | 17 | 18.441816 | Excluded |
| rs47706937 | 17 | 18.597440 | 10.645047 |
| rs49889931 | 17 | 18.738050 | 10.645047 |
| rs3669693 | 17 | 18.890202 | Excluded |
| rs51833596 | 17 | 19.196246 | 10.645047 |
| rs49322419 | 17 | 19.347875 | 10.645047 |
| rs48658548 | 17 | 19.498627 | 10.645047 |
| rs33623925 | 17 | 19.638402 | Excluded |
| rs33443991 | 17 | 19.761457 | Excluded |
| rs33313219 | 17 | 19.883511 | Excluded |
| rs46896677 | 17 | 20.005655 | 11.732731 |
| rs45930193 | 17 | 20.124206 | Excluded |
| rs33505791 | 17 | 20.250750 | 12.820416 |
| rs46758914 | 17 | 20.344891 | 12.820416 |
| rs50616914 | 17 | 20.414578 | Excluded |
| rs51793448 | 17 | 20.556905 | 12.820416 |
| rs45892529 | 17 | 20.639924 | 12.820416 |
| rs50350961 | 17 | 20.708003 | Excluded |
| rs33558668 | 17 | 20.873043 | 12.820416 |
| rs50599938 | 17 | 21.026737 | 12.820416 |
| rs49578742 | 17 | 21.131488 | 12.820416 |
| rs46456736 | 17 | 21.269569 | Excluded |
| rs6309667 | 17 | 21.399470 | 12.820416 |
| rs51776929 | 17 | 21.451856 | 12.820416 |
| rs51519665 | 17 | 21.570619 | 12.820417 |
| rs47071440 | 17 | 21.647620 | Excluded |
| rs47597775 | 17 | 21.704163 | 12.820417 |
| rs46816700 | 17 | 21.798593 | 12.820417 |
| rs48587745 | 17 | 21.884244 | 13.192429 |
| rs47656704 | 17 | 21.982674 | 13.192429 |
| rs51362341 | 17 | 22.064371 | 13.192430 |
| rs47632254 | 17 | 22.129407 | Excluded |
| rs33894607 | 17 | 22.218269 | 13.192430 |
| rs46464081 | 17 | 22.302933 | Excluded |
| rs33768528 | 17 | 22.423324 | Excluded |
| rs33768715 | 17 | 22.511837 | 13.192430 |
| rs6380479 | 17 | 22.593277 | 13.192430 |
| rs33762862 | 17 | 22.643379 | 13.192430 |
| rs33761921 | 17 | 22.742107 | Excluded |
| rs33761713 | 17 | 22.826175 | 13.192430 |
| rs31227446 | 17 | 22.918955 | 13.192430 |
| rs51876982 | 17 | 23.038637 | 13.192430 |
| rs51286236 | 17 | 23.152019 | 13.192430 |
| rs52262915 | 17 | 23.292930 | Excluded |
| rs33650594 | 17 | 23.328663 | 13.192430 |
| rs50419142 | 17 | 23.447648 | 13.192431 |
| rs46975453 | 17 | 23.591855 | 13.192431 |
| rs47393377 | 17 | 23.742906 | 13.192431 |
| rs4231351 | 17 | 23.819207 | 13.192431 |
| rs29518971 | 17 | 23.890474 | Excluded |
| rs51336104 | 17 | 23.993879 | 13.564456 |
| rs48701347 | 17 | 24.109659 | 13.749505 |
| rs51090517 | 17 | 24.229772 | 14.120786 |
| rs29524838 | 17 | 24.230479 | Excluded |
| rs45868248 | 17 | 24.302236 | 14.120786 |
| rs49802776 | 17 | 24.401276 | 14.120786 |
| rs47797592 | 17 | 24.503324 | 14.120786 |
| rs47690895 | 17 | 24.592244 | 14.120786 |
| rs48951432 | 17 | 24.705655 | 14.120787 |
| rs33568823 | 17 | 24.779303 | 14.120787 |
| rs13465803 | 17 | 24.877096 | 14.120787 |
| rs13473530 | 17 | 24.986443 | 14.120787 |
| rs3155840 | 17 | 25.017092 | 14.120787 |
| rs3154010 | 17 | 25.051642 | 14.120787 |
| rs33778632 | 17 | 25.185444 | 14.120787 |
| rs3720371 | 17 | 25.307057 | 14.305856 |
| rs33311501 | 17 | 25.435339 | Excluded |
| rs33776207 | 17 | 25.524432 | 14.305856 |
| rs33774939 | 17 | 25.627780 | 14.491298 |
| rs51588295 | 17 | 25.707243 | 14.676364 |
| rs49591784 | 17 | 25.859131 | Excluded |
| rs49255793 | 17 | 25.952787 | Excluded |
| rs13473224 | 17 | 25.986908 | 14.676364 |
| rs50622796 | 17 | 26.020331 | 14.676364 |
| rs49624859 | 17 | 26.066076 | 14.676364 |
| rs8252810 | 17 | 26.101225 | 14.676364 |
| rs47909630 | 17 | 26.218812 | 14.676364 |
| rs48788115 | 17 | 26.325583 | 14.676364 |
| rs48620320 | 17 | 26.350407 | 14.676365 |
| rs33696562 | 17 | 26.420086 | 14.676365 |
| rs48790914 | 17 | 26.581905 | 14.676365 |
| rs47632501 | 17 | 26.679592 | 14.676365 |
| rs49986009 | 17 | 26.792980 | 14.861432 |
| rs48187606 | 17 | 26.879445 | 14.861432 |
| rs47820345 | 17 | 26.929046 | Excluded |
| rs47161053 | 17 | 27.075018 | 15.046115 |
| rs51492876 | 17 | 27.112142 | 15.046115 |
| rs50422773 | 17 | 27.235278 | 15.415867 |
| rs33797547 | 17 | 27.327056 | 15.415867 |
| rs33793841 | 17 | 27.393948 | 15.415867 |
| rs33796995 | 17 | 27.485137 | 15.415867 |
| rs6397584 | 17 | 27.588410 | 16.543696 |
| rs33797855 | 17 | 27.639775 | 16.543696 |
| rs33793002 | 17 | 27.717750 | 16.543696 |
| rs33788824 | 17 | 27.796735 | Excluded |
| rs33791811 | 17 | 27.889877 | 16.543696 |
| rs33421366 | 17 | 28.013769 | 16.543696 |
| rs33787061 | 17 | 28.145985 | 16.543696 |
| rs33788207 | 17 | 28.290598 | 16.543697 |
| rs49085856 | 17 | 28.451469 | 17.099940 |
| rs46306385 | 17 | 28.577926 | 17.099940 |
| rs46583703 | 17 | 28.695754 | 17.099940 |
| rs48729540 | 17 | 28.731145 | 17.099940 |
| rs47414840 | 17 | 28.821694 | 17.099940 |
| rs46530666 | 17 | 28.947581 | 17.099940 |
| rs33145938 | 17 | 29.018310 | 17.099940 |
| rs49828597 | 17 | 29.040229 | 17.099940 |
| rs47431288 | 17 | 29.108910 | 17.099940 |
| rs16813263 | 17 | 29.235365 | 17.099941 |
| rs50622422 | 17 | 29.295908 | 17.099941 |
| rs50417476 | 17 | 29.400834 | Excluded |
| rs51264774 | 17 | 29.438389 | 17.099941 |
| rs45986438 | 17 | 29.515281 | 17.099941 |
| rs50551482 | 17 | 29.631575 | 17.099941 |
| rs48332629 | 17 | 29.705469 | 17.284931 |
| rs50660359 | 17 | 29.813195 | 17.284931 |
| rs46891381 | 17 | 29.966212 | Excluded |
| rs48922983 | 17 | 30.077779 | 17.284931 |
| rs33805377 | 17 | 30.136032 | 17.284931 |
| rs33798932 | 17 | 30.345006 | Excluded |
| rs13482943 | 17 | 30.436490 | 17.284931 |
| rs33796254 | 17 | 30.667147 | 17.284931 |
| rs47702132 | 17 | 30.729822 | 17.284932 |
| rs3150650 | 17 | 30.849242 | Excluded |
| rs49113180 | 17 | 30.940109 | Excluded |
| rs46367947 | 17 | 31.092097 | Excluded |
| rs33345493 | 17 | 31.235242 | 17.656796 |
| rs47336736 | 17 | 31.262610 | 17.656796 |
| rs47100788 | 17 | 31.390112 | 17.656796 |
| rs48620478 | 17 | 31.437384 | Excluded |
| rs51849267 | 17 | 31.580828 | 17.656796 |
| rs51912574 | 17 | 31.664500 | 17.656797 |
| rs29501189 | 17 | 31.754198 | 17.656797 |
| rs51696841 | 17 | 31.815014 | 17.656797 |
| rs45936635 | 17 | 31.984352 | 18.031981 |
| rs48227067 | 17 | 32.083558 | 18.031981 |
| rs47231957 | 17 | 32.188698 | 18.031981 |
| rs33175238 | 17 | 32.281533 | 18.031982 |
| rs45775679 | 17 | 32.376141 | Excluded |
| rs46378993 | 17 | 32.441111 | 18.031982 |
| rs46437881 | 17 | 32.629686 | 18.031982 |
| rs51609585 | 17 | 32.772095 | 18.031982 |
| rs51650057 | 17 | 32.808023 | 18.031982 |
| rs46683460 | 17 | 32.908696 | 18.031982 |
| rs48824806 | 17 | 33.016256 | Excluded |
| rs48896493 | 17 | 33.066955 | 18.031982 |
| rs49307866 | 17 | 33.114279 | Excluded |
| rs49206939 | 17 | 33.156445 | Excluded |
| rs33387399 | 17 | 33.204560 | 18.031982 |
| rs33599214 | 17 | 33.283941 | 18.031982 |
| rs49775540 | 17 | 33.408078 | 18.031982 |
| rs33167198 | 17 | 33.471662 | 18.031983 |
| rs46894227 | 17 | 33.517890 | 18.031983 |
| rs48850739 | 17 | 33.605592 | 18.031983 |
| rs46588791 | 17 | 33.688095 | 18.031983 |
| rs3724223 | 17 | 33.713387 | Excluded |
| rs33399614 | 17 | 33.781645 | 18.219725 |
| rs13466777 | 17 | 33.814309 | 18.219725 |
| rs33219958 | 17 | 33.882315 | 18.219725 |
| rs33473732 | 17 | 33.985431 | 18.219725 |
| rs13461487 | 17 | 34.080384 | 18.219725 |
| rs50664936 | 17 | 34.201052 | 18.596365 |
| rs46967024 | 17 | 34.320037 | 19.936483 |
| rs51014158 | 17 | 34.380461 | 19.936483 |
| rs51322511 | 17 | 34.420628 | 19.936484 |
| rs46657641 | 17 | 34.518446 | 19.936484 |
| rs33137036 | 17 | 34.553437 | 19.936484 |
| rs33352225 | 17 | 34.719858 | 20.123432 |
| rs47857138 | 17 | 34.819459 | 20.123432 |
| rs49021161 | 17 | 34.997862 | 20.123432 |
| rs49284945 | 17 | 35.058663 | 20.123432 |
| rs51566712 | 17 | 35.189788 | 20.497724 |
| rs49458120 | 17 | 35.203712 | 20.497725 |
| rs46751650 | 17 | 35.250498 | 20.872012 |
| rs33128146 | 17 | 35.374902 | 20.872012 |
| rs50942026 | 17 | 35.460431 | Excluded |
| rs51364603 | 17 | 35.576619 | Excluded |
| rs50609445 | 17 | 35.680403 | 20.872012 |
| rs29539664 | 17 | 35.704867 | Excluded |
| rs50991099 | 17 | 35.773832 | 20.872012 |
| rs50915559 | 17 | 35.827011 | 20.872012 |
| rs51376240 | 17 | 35.948190 | 20.872012 |
| rs33244455 | 17 | 36.088559 | Excluded |
| rs33536488 | 17 | 36.100416 | 20.872012 |
| rs48799614 | 17 | 36.151588 | 20.872012 |
| rs46978576 | 17 | 36.275311 | 20.872013 |
| rs50486010 | 17 | 36.328396 | 20.872013 |
| rs33336270 | 17 | 36.502331 | 20.872013 |
| rs50127319 | 17 | 36.656456 | 20.872013 |
| rs49166645 | 17 | 36.808602 | 20.872013 |
| rs47431559 | 17 | 36.989510 | 20.872013 |
| rs47224170 | 17 | 37.125945 | Excluded |
| rs33354969 | 17 | 37.209139 | 20.872013 |
| rs47681519 | 17 | 37.334983 | 20.872013 |
| rs49926056 | 17 | 37.407556 | 20.872013 |
| rs47663051 | 17 | 37.542510 | 20.872013 |
| rs33263595 | 17 | 37.620392 | 20.872014 |
| rs33721754 | 17 | 37.781068 | 20.872014 |
| rs3145660 | 17 | 37.942794 | 20.872014 |
| rs33266489 | 17 | 38.059976 | Excluded |
| rs33809121 | 17 | 38.344545 | 20.872014 |
| rs33809115 | 17 | 38.412326 | 20.872014 |
| rs33808113 | 17 | 38.781141 | 20.872014 |
| rs46784111 | 17 | 39.120897 | 20.872014 |
| rs48863565 | 17 | 39.500366 | Excluded |
| rs33719543 | 17 | 39.855918 | 20.872014 |
| rs50466635 | 17 | 40.555729 | 20.872014 |
| rs48843536 | 17 | 40.570888 | Excluded |
| rs33818802 | 17 | 40.771011 | 21.438242 |
| rs33510216 | 17 | 40.862078 | 21.438242 |
| rs33717366 | 17 | 40.927587 | 21.438243 |
| rs3703171 | 17 | 41.098433 | 21.438243 |
| rs29504333 | 17 | 41.281725 | 21.438243 |
| rs33814666 | 17 | 41.398561 | 21.438243 |
| rs33810270 | 17 | 41.669826 | 21.438243 |
| rs47337390 | 17 | 41.968304 | 21.438243 |
| rs51198846 | 17 | 42.059392 | 21.438243 |
| rs33341718 | 17 | 42.257203 | 23.183752 |
| rs50798438 | 17 | 42.546241 | 23.183752 |
| rs29522360 | 17 | 42.639529 | 23.183752 |
| rs47388201 | 17 | 42.729732 | 23.183752 |
| rs47895662 | 17 | 42.804633 | 23.183752 |
| rs52170358 | 17 | 42.952087 | 23.183753 |
| rs47222504 | 17 | 43.176984 | 23.183753 |
| rs4231500 | 17 | 43.225906 | 23.183753 |
| rs33439875 | 17 | 43.326565 | 23.183753 |
| rs47356867 | 17 | 43.432112 | 23.183753 |
| rs49158050 | 17 | 43.559462 | 23.183753 |
| rs48263700 | 17 | 43.615176 | 23.183753 |
| rs49149214 | 17 | 43.616329 | 23.183753 |
| rs51295335 | 17 | 43.739797 | 23.183753 |
| rs49026999 | 17 | 43.763410 | 23.183753 |
| rs47286629 | 17 | 43.817111 | 23.183754 |
| rs49197890 | 17 | 43.888149 | 23.183754 |
| rs33361028 | 17 | 44.091014 | 23.183754 |
| rs51254854 | 17 | 44.158045 | 23.183754 |
| rs48036527 | 17 | 44.218396 | 23.183754 |
| rs50448870 | 17 | 44.236328 | 23.183754 |
| rs29498067 | 17 | 44.338085 | 23.183754 |
| rs33057183 | 17 | 44.412754 | 23.183754 |
| rs49233337 | 17 | 44.511617 | 23.183754 |
| rs49904346 | 17 | 44.695930 | Excluded |
| rs49223069 | 17 | 44.795948 | Excluded |
| rs33391756 | 17 | 44.853609 | 23.372533 |
| rs50774704 | 17 | 44.980412 | 23.372533 |
| rs33246586 | 17 | 45.104239 | 23.372533 |
| rs46074101 | 17 | 45.262851 | 23.372533 |
| rs50650188 | 17 | 45.361219 | Excluded |
| rs49336210 | 17 | 45.465625 | Excluded |
| rs50562643 | 17 | 45.545315 | Excluded |
| rs49209168 | 17 | 45.645495 | 23.561312 |
| rs13474307 | 17 | 45.723200 | Excluded |
| rs48762654 | 17 | 45.868876 | 23.745648 |
| rs51288380 | 17 | 46.016810 | Excluded |
| rs16796521 | 17 | 46.162476 | Excluded |
| rs46927280 | 17 | 46.281127 | Excluded |
| rs29497596 | 17 | 46.351150 | Excluded |
| rs50974076 | 17 | 46.449416 | Excluded |
| rs49444046 | 17 | 46.548114 | Excluded |
| rs47018538 | 17 | 46.645369 | Excluded |
| rs49710933 | 17 | 46.723242 | Excluded |
| rs33429825 | 17 | 46.823618 | Excluded |
| rs13474855 | 17 | 46.911525 | Excluded |
| rs49276771 | 17 | 47.028372 | Excluded |
| rs49491100 | 17 | 47.103117 | Excluded |

SNP markers and their chromosomal positions according to <http://genome.ucsc.edu>. Mm9 sequence was obtained from the Build 37 assembly by NCBI and the Mouse Genome Sequencing Consortium. Excluded, markers not used in the analysis.
