# Supplementary material for: High-Resolution Mapping of a Genetic Locus Regulating Preferential Carbohydrate Intake, Total Kilocalories, and Food Volume on Mouse Chromosome 17
Source: PLoS One. 2014 Oct 20;9(10):e110424. doi: 10.1371/journal.pone.0110424 (PMC4203797; doi:10.1371/journal.pone.0110424)
Supplement: Table S3 — Correlation matrix for nutrient intake phenotypes and post-diet selection characteristics in the recombinant congenic F2 population. Legend: Carbohydrate/protein (C/P), fat/protein (F/P), total kilocalories (kcal) and total food volume (g) consumed; body weight (BW 2), body weight gain from BW 1 to BW 2; body fat (NMR2 fat) and body lean mass (NMR2 lean) after 10 d macronutrient diet selection; fat mass gain (Fat gain 12) and lean mass gain (lean gain12) over 10 d diet selection. a P<0.05; b P<0.01; c P<0.001, d P<0.0001. (DOCX) [file pone.0110424.s007.docx]

Table S3. Correlation matrix for nutrient intake phenotypes and post-diet selection characteristics in the recombinant congenic F_2_ population.

|  | *C/P kcal* | *F/P kcal* | *Total kcal* | *Total food volume (g)* | *BW 2 (g)* | *BW Gain 12* | *NMR2 fat* | *NMR2 lean* | *Fat Gain 12* | *Lean Gain 12* |
| --- | --- | --- | --- | --- | --- | --- | --- | --- | --- | --- |
| *C/P kcal* | 1.00 | -0.78^d^ | 0.46^d^ | 0.86^d^ | 0.17^a^ | 0.12 | 0.10 | 0.13 | 0.18^a^ | -0.85 |
| *F/P kcal* |  | 1.00 | 0.19^a^ | -0.36^d^ | 0.23^b^ | 0.22^b^ | 0.23^b^ | 0.22^b^ | 0.22^b^ | 0.24^b^ |
| *Total kcal* |  |  | 1.00 | 0.85^d^ | 0.60^d^ | 0.51^d^ | 0.48^d^ | 0.52^d^ | 0.60^d^ | 0.21^b^ |
| *Total food volume (g)* |  |  |  | 1.00 | 0.45^d^ | 0.36^d^ | 0.33^d^ | 0.37^d^ | 0.45^d^ | 0.07 |
| *BW 2 (g)* |  |  |  |  | 1.00 | 0.67^d^ | 0.79^d^ | 0.79^d^ | 0.64^d^ | 0.32^a^ |
| *BW Gain 12* |  |  |  |  |  | 1.00 | 0.69^d^ | 0.43^d^ | 0.70^d^ | 0.41^d^ |
| *NMR2 fat* |  |  |  |  |  |  | 1.00 | 0.63^d^ | 0.82^d^ | 0.38^d^ |
| *NMR2 lean* |  |  |  |  |  |  |  | 1.00 | 0.60^d^ | 0.49^d^ |
| *Fat Gain 12* |  |  |  |  |  |  |  |  | 1.00 | 0.40^d^ |
| *Lean Gain 12* |  |  |  |  |  |  |  |  |  | 1.00 |

Carbohydrate/protein (C/P), fat/protein (F/P), total kilocalories (kcal) and total food volume (g) consumed; body weight (BW 2), body weight gain from BW 1 to BW 2; body fat (NMR2 fat) and body lean mass (NMR2 lean) after 10 d macronutrient diet selection; fat mass gain (Fat gain 12) and lean mass gain (lean gain12) over 10 d diet selection. ^a^*P* < 0.05; ^b^*P* < 0.01; ^c^*P* < 0.001, ^d^*P*<0.0001.
