# Supplementary material for: High-Resolution Mapping of a Genetic Locus Regulating Preferential Carbohydrate Intake, Total Kilocalories, and Food Volume on Mouse Chromosome 17
Source: PLoS One. 2014 Oct 20;9(10):e110424. doi: 10.1371/journal.pone.0110424 (PMC4203797; doi:10.1371/journal.pone.0110424)
Supplement: Table S4 — Correlation matrix for nutrient intake phenotypes and baseline, pre-diet selection characteristics in the non-recombinant congenic F2 population. Legend: Carbohydrate/protein (C/P), fat/protein (F/P), total kilocalories (kcal) and total food volume (g) consumed; body weight (BW 1), body fat (NMR1 fat), and body lean mass (NMR1 lean) when diet selection began. a P<0.05; b P<0.01; c P<0.001, d P<0.0001. (DOCX) [file pone.0110424.s008.docx]

Table S4. Correlation matrix for nutrient intake phenotypes and baseline, pre-diet selection characteristics in the non-recombinant congenic F_2_ population

|  | *C/P kcal* | *F/P kcal* | *Total kcal* | *Total food volume (g)* | *BW 1 (g)* | *NMR1 fat (g)* | *NMR1 lean (g)* |
| --- | --- | --- | --- | --- | --- | --- | --- |
| *C/P kcal* | 1.00 | -0.81^d^ | 0.46^d^ | 0.87^d^ | 0.15 | -0.14 | 0.20^a^ |
| *F/P kcal* |  | 1.00 | 0.13 | -0.43^d^ | 0.08 | 0.12 | 0.00 |
| *Total kcal* |  |  | 1.00 | 0.83^d^ | 0.39^d^ | -0.05 | 0.36^d^ |
| *Total food volume (g)* |  |  |  | 1.00 | 0.31^d^ | -0.11 | 0.32^d^ |
| *BW 1 (g)* |  |  |  |  | 1.00 | 0.35^d^ | 0.89^d^ |
| *NMR1 fat (g)* |  |  |  |  |  | 1.00 | 0.07^b^ |
| *NMR1 lean (g)* |  |  |  |  |  |  | 1.00 |

Carbohydrate/protein (C/P), fat/protein (F/P), total kilocalories (kcal) and total food volume (g) consumed; body weight (BW 1), body fat (NMR1 fat), and body lean mass (NMR1 lean) when diet selection began. ^a^*P* < 0.05; ^b^*P* < 0.01; ^c^*P* < 0.001, ^d^*P*<0.0001.
