# Supplementary material for: High-Resolution Mapping of a Genetic Locus Regulating Preferential Carbohydrate Intake, Total Kilocalories, and Food Volume on Mouse Chromosome 17
Source: PLoS One. 2014 Oct 20;9(10):e110424. doi: 10.1371/journal.pone.0110424 (PMC4203797; doi:10.1371/journal.pone.0110424)
Supplement: Table S6 — Body weight and composition data for HQ17IIa sub-congenic mice. Legend: Values (mean ± SE) were obtained immediately before and then after the 10 d period of macronutrient diet selection. (DOCX) [file pone.0110424.s010.docx]

Table S6. Body weight and composition data for HQ17IIa

sub-congenic mice.

|  | Subcongenic  (n = 14) | Wild type  (n = 13) |
| --- | --- | --- |
| Body weight (g)  Before diet  After diet | 35.5 ± 0.5  36.8 ± 0.4 | 36.8 ± 0.7  37.9 ± 0.8 |
| % Fat mass  Before diet  After diet | 7.5 ± 0.6  11.2 ± 0.8 | 7.9 ± 0.7  11.1 ± 1.1 |
| % Muscle mass  Before diet  After diet | 76.9 ± 0.4  71.2 ± 0.6 | 75.9 ± 0.6  69.6 ± 1.7 |
| % Fluid  Before diet  After diet | 12.5 ± 0.1  12.1 ± 0.1 | 12.4 ± 0.1  12.4 ± 0.1 |

Values (mean ± S.E.M.) were obtained before and after the 10 d period of macronutrient diet selection.
